# Anti-Inflammatory Constituents from *Bidens frondosa*

**DOI:** 10.3390/molecules201018496

**Published:** 2015-10-09

**Authors:** Jiamei Le, Wenquan Lu, Xiaojuan Xiong, Zhijun Wu, Wansheng Chen

**Affiliations:** 1 Department of Pharmacy, Changzheng Hospital, Second Military Medical University, Shanghai 200003, China; E-Mails: lejiamei0923@163.com (J.L.); lwqp@163.com (W.L.); 2 College of Chemical and Biological Engineering, Yichun University, Jiangxi 336000, China; E-Mail: ycxxxj@163.com

**Keywords:** *Bidensfrondosa*, polyacetylene glucoside, phenylpropanoid glucoside, flavonoid glycoside, anti-inflammatory activity

## Abstract

A new polyacetylene glucoside (3*E*,5*E*,11*E*)-tridecatriene-7,9-diyne-1,2,13-triol-2-*O*-β-d-glucopyranoside (**1**), a new phenylpropanoid glucoside 2′-butoxyethylconiferin (**2**), and a new flavonoid glycoside 8,3′,4′-trihydroxyflavone-7-*O*-(6′′-*O*-*p*-coumaroyl)-β-d-glucopyranoside (**3**), have been isolated from *Bidens frondosa* together with fifty-three known compounds **4**–**56**. The structures of these compounds were established by spectroscopic methods. mainly ESIMS, 1D- and 2D-NMR spectroscopic data. and comparison with literature data. Compounds **1**–**34**, **36**, **39**, **43**, **47**, **51**, and **52** were tested for inhibition of nuclear factor kappa B (NF-κB) in 293-NF-κB-luciferase report cell line induced by lipopolysaccharide (LPS), and compounds **1**, **2**, **3**, **9**, **15**, **21**, **24** and **51** were tested for the production of TNF-α, IL-1β, IL-6, IL-10 in RAW 264.7 macrophages induced by LPS. In conclusion, the isolated compounds **1**, **2**, **3**, **9**, **15**, **21**, **24** and **51** exhibited significant activity in anti-inflammatory activity assays.

## 1. Introduction

*Bidens* *frondosa* (L.) is an annual herbaceous plant growing widely on nutrient-rich mudsoils or muddy sandsoils at the shores of rivers and lakes, which is one of species of genus *Bidens* from the family Asteraceae. It is native to North America, and now distributed throughout China. It has attracted a great deal of attention for its wide range of biological activities, such as antibacterial [1], antioxidant [2], antimalarial [3] and antidiarrheal [4]. Previous studies reported that *B. frondosa* contained acetylene [3], polyenic D-glucosides [5,6], chalcones and aurones [2,3,7,8], flavones and terpenes [9]. However, a literature survey reveals that there are no reports on the anti-inflammatory activity of *B.* *frondosa*, and the research on chemical constituents of *B.* *frondosa* is also limited. Thus we decided to isolate chemical constituents of *B.* *frondosa* and to investigate its anti-inflammatory activity.

As part of an ongoing search for bioactive natural products from traditional folk medicines, we have conducted a phytochemical investigation on the EtOH extract of *B.* *frondosa*. This has led to the isolation of a new polyacetylene glucoside, a new phenylpropanoid glucoside, a new flavonoid glycoside and fifty-three known compounds, identified as (3*E*,5*E*,11*E*)-tridecatriene-7,9-diyne-1,2,13-triol-2-*O*-β-d-glucopyranoside (**1**), 2′-butoxyethylconiferin (**2**), 8,3′,4′-trihydroxyflavone-7-*O*-(6′′-*O*-*p*-coumaroyl)-β-d-glucopyranoside (**3**), butylconiferin (**4**), 2-methoxy-4-(2-propenyl)-phenyl-β-d-glucopyranoside (**5**), 2-methoxy-4-(2′-hydroxyethyl)-phenol-1-*O*-β-d-glucopyranoside (**6**), (1′*R*,2′*R*)-guaiacyl glycerol 3′-*O*-β-d-glucopyranoside (**7**), *threo*-5-hydroxy-3,7-dimethoxyphenylpropane-8,9-diol (**8**), 3-(4-hydroxy-3-methoxyphenyl)-3-methoxypropane-1,2-diol (**9**), 3-(4-hydroxy-3-methoxy-phenyl)-propane-1,2-diol (**10**), guaiacylglycerol (**11**), ananasate (**12**), *p*-hydroxyphenyl-6-*O*-*trans*-caffeoyl-β-d-alloside (1**3**), 6′-*O*-caffeoyl-*p*-hydroxyacetophenone-4-*O*-β-d-glucopyranoside (**14**), wilfordiol B (**15**), 4,5-di-*O*-caffeoylquinic acid 1-methyl ether (**16**), caffeoylcalleryanin (**17**), 1-*O*-(*E*)-caffeoyl-β-d-gentiobiose (**18**), plantasioside (**19**), okanin-4′-*O*-(6′′-*O*-*p*-coumaroyl-β-d-glucopyranoside) (**20**), (−)-4′-methoxy-7-*O*-(6′′-acetyl)-β-d-glucopyranosyl-8,3′-dihydroxyflavanone (**21**), (−)-4′-methoxy-7-*O*-β-d-glucopyranosyl-8,3′-dihydroxyflavanone (**22**), hesperetin-7-*O*-β-d-glucopyranoside (**23**), apigenin (**24**), 3′-hydroxyscutellarein-7-*O*-(6′′-*O*-protocatechuoyl)-β-glucopyranoside (**25**), quercetin-3-*O*-glucopyranoside (**26**), 8,3′,4′-trihydroxyflavone-7-*O*-β-d-glucopyranoside (**27**), luteolin-7-*O*-glucoside (**28**), 6-hydroxyluteolin-7-*O*-glucoside (**29**), 3′′-(3-hydroxy-3-methyl-glutaroyl)-ester of 6-hydroxy-luteolin-7-*O*-β-d-glucopyranoside (**30**), luteolin-7-*O*-(β-d-glucopyranosyl)-2-glucopyranoside (**31**), (*Z*)-7-*O*-β-d-glucopyranosyl-6,7,3′,4′-tetrahydroxyaurone (**32**), sulfuretin-6-*O*-β-d-glucoside (**33**), maritimetin-6-*O*-β-d-glucoside (**34**), dihydrophaseic acid (**35**), caffeic acid (**36**), isoferuloyl ethyl ester (**37**), protocatechuic acid (**38**), 1,3,5-trimethoxybenzene (**39**), vanillin (**40**), 7*R*,11*R*-phytol (**41**), 1-octacosanol (**42**), indole-3-carboxylic acid (**43**), 1*H*-indole-3-carboxadehyde (**44**), niacinamide (**45**), β-sitosterol (**46**), stigmasterol (**47**), hiziprafuran (**48**), 5-hydroxy-2-furaldehyde (**49**), α-tocopherol (**50**), 4-hydroxy-2-furaldehyde (**51**), α-tocospiro A (**52**), ethyl linoleate (**53**), methyl linolenate (**54**), tripalmitolein (**55**) and trilinolenin (**56**). Compounds **1**–**34**, **36**, **39**, **43**, **47**, **51**, and **52** were tested for anti-inflammatory activities, and several of the isolated compounds (**1**, **2**, **3**, **9**, **15**, **21**, **24** and **51**) exhibited significant activities in anti-inflammatory activity assays. Here, we report the isolation of these compounds, structure elucidation of three new compounds and anti-inflammatory activities of compounds **1**, **2**, **3**, **9**, **15**, **21**, **24** and **51**.

## 2. Results and Discussion

The 80% EtOH extract of air-dried *B.* *frondosa* was suspended in H_2_O and partitioned successively with petroleum ether, EtOAc and *n*-BuOH, respectively. The petroleum ether, EtOAc and *n*-BuOH fractions were then purified repeatedly by column chromatography with silica gel, Sephadex LH-20 and ODS C18 to yield compounds **1**–**56**.

### 2.1. Structural Elucidation of Isolated Compounds

**Figure 1:**
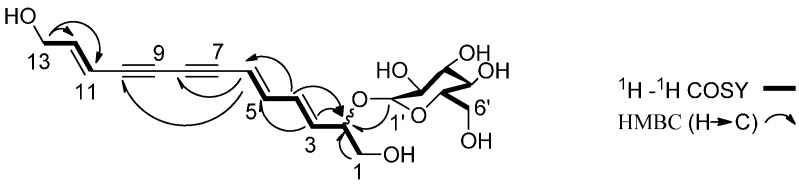
Key ^1^H-^1^H COSY and HMBC (H→C) correlations of compound **1**.

Compound **1** was isolated as a brownish amorphous powder. The positive HR-ESIMS showed a pseudo-molecular ion at *m*/*z* 403.1371 [M + Na]^+^ (calcd. for C_19_H_24_O_8_Na, 403.1369), consistent with the formula C_19_H_24_O_8_, indicating the presence of eight degrees of unsaturation. The IR spectrum showed absorptions due to hydroxy (3362 cm^−1^) and olefinic bond (1658, 1632 cm^−1^) moieties. The ^13^C-NMR spectrum exhibited 19 carbon resonances, classified into four quaternary carbons, twelve methines including five oxygen-substituted carbons, and three oxygen-substituted methylenes. By analysis of degrees of unsaturation, four quaternary carbon signals at δ_C_ 81.9, 81.5, 76.2, 73.8 were ascribed to two acetylene bonds. The signals at δ_C_ 149.5, 145.5, 137.9, 129.8, 109.1, 106.4 were ascribed to three double bonds. The signals at δ_C_ 102.7, 77.1, 76.8, 74.0, 70.2, 61.0 were assigned to a glucopyranosyl unit. In the ^1^H-NMR spectrum, the signals at δ_H_ 6.81 (1H, dd, *J* = 15.6, 10.8 Hz), 6.48 (1H, ddd, *J* = 16.2, 9.0, 8.4 Hz), 6.41 (1H, dd, *J* = 15.6, 10.8 Hz), 6.00 (1H, dd, *J* = 15.6, 5.4 Hz), 5.88 (1H, d, *J* = 15.6 Hz), 5.86 (1H, d, *J* = 16.2 Hz) implied the presence of three conjugated double bonds with *E* geometry as deduced from the coupling constants [10]. The ^1^H- and ^13^C-NMR spectra of **1** were quite similar to those of (2*E*,8*E*,10*E*)-tridecatriene-4,6-diyne-1,12,13-triol-1-*O*-β-d-glucopyranoside (**1a**) [11] (Figure S1). A comparison of its spectrum with **1a** showed that the glucopyranosyl unit was linked at C-2 in compound **1** instead of C-1 in compound **1a**. HMBC correlation from H-1′ (δ_H_ 4.27, d, *J* = 7.8 Hz) to C-2 (δ_C_ 79.9) confirmed the glucopyranosyl unit was linked at C-2. The configuration of C-2 remained undetermined. Therefore, the structure of **1** was elucidated as (3*E*,5*E*,11*E*)-tridecatriene-7,9-diyne-1,2,13-triol-2-*O-*β-d-glucopyranoside (Figure 1).

**Figure 2:**
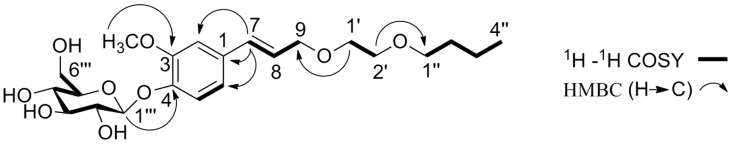
Key ^1^H-^1^H COSY and HMBC (H→C) correlations of compound **2**.

Compound **2** was isolated as a white amorphous powder. The negative HR-ESIMS showed a pseudo-molecular ion at *m*/*z* 477.1917 [M + Cl]^−^ (calcd for C_22_H_34_O_9_Cl, 477.1891), consistent with the formula C_22_H_34_O_9_, indicating the presence of six degrees of unsaturation. The ^13^C-NMR spectrum exhibited 22 carbon resonances, classified into three quaternary carbons, ten methines including four oxygen-substituted carbons, seven methylenes including five oxygen-substituted carbons, one methyl group and one methoxyl group. By analysis of degrees of unsaturation, the signals at δ_C_ 133.3, 125.9 were ascribed to a double bond and the signals at δ_C_ 150.9, 147.8, 133.4, 120.9, 117.9, 111.4 were ascribed to an aromatic ring. The DEPT spectrum showed signals at δ_C_ 71.2, 70.5 and 72.1, 32.8, 20.3, 14.2 that were ascribed to a -*O*-*C*-1′-*C*-2′-*O*- diether chain and a terminal -*O*-*C*-1′′-*C*-2′′-*C*-3′′-*C*-4′′ chain, respectively The signals at δ_C_ 102.7, 78.2, 77.8, 74.9, 71.3, 62.5 were assigned to a glucopyranosyl unit. In the ^1^H-NMR spectrum, the signals at δ_H_ 6.54 (1H, d, *J* = 15.6 Hz), 6.20 (1H, dd, *J* = 15.6, 6.0 Hz) implied the presence of a conjugated double bond with *E* geometry as deduced from the coupling constants. The signals at δ_H_ 7.06 (1H, d, *J* = 8.4 Hz), 7.03 (1H, d, *J* = 1.8 Hz), 6.90 (1H, dd, *J* = 8.4, 1.8 Hz) showed an ABX aromatic ring spin system and implied the presence of 1,3,4-trisubstituted aromatic ring [10]. The ^1^H- and ^13^C-NMR spectra of **2** were quite similar to those of ethylconiferin (**2a**) [12] (Figure S1). Detailed comparison of the NMR data of **2** with **2a** indicated that there was an additional butoxy group in compound **2**. In the HMBC spectrum of **2**, a correlation from H-2′ (δ_H_ 3.56) to the C-1′′ (δ_C_ 72.1) position of the butoxy was observed, which suggested the butoxy was linked at C-2′. The structure of **2** was established on the basis of 1D- and 2D-NMR spectra (^1^H, ^13^C, DEPT, COSY, HSQC and HMBC). Accordingly, the structure of compound **2** was elucidated as 2′-butoxyethylconiferin (Figure 2). In addition, we have found compound **2** was present in a crude extract before contact with butanol, which was tested by HPLC (Figure S24). From HR-ESIMS, 1D- and 2D-NMR spectroscopic data (Figure S9–S15) and the HPLC profile, we think that compound **2** is a natural chemical constituent and not an isolation artefact. 

**Figure 3:**
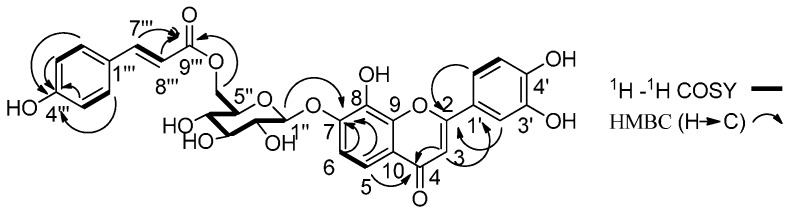
Key ^1^H-^1^H COSY and HMBC (H→C) correlations of compound **3**.

Compound **3** was isolated as a brownish-yellow amorphous powder. The negative HR-ESIMS showed a pseudo-molecular ion at *m*/*z* 593.1316 [M − H]^−^ (calcd for C_30_H_25_O_13_, 593.1295), consistent with the formula C_30_H_26_O_13_, indicating the presence of 18 degrees of unsaturation. The IR spectrum showed absorptions due to hydroxy (3405 cm^−1^), aromatic ring (1513, 1443 cm^−1^) and olefinic bonds (1688, 1649 cm^−1^). The ^13^C-NMR spectrum exhibited 30 carbon resonances, classified into twelve quaternary carbons, seventeen methines including four oxygen-substituted carbons, one oxygen-substituted methylenes. The signals at δ_C_ 182.6, 154.4, 152.3, 145.8, 132.8, 117.4, 114.4, 113.2, 112.0 were ascribed to a flavonoids skeleton. The signals at δ_C_ 166.7, 160.2, 145.2, 130.6, 125.3, 116.1, 114.2 were ascribed to a *p*-coumaroyl group. The signals at δ_C_ 101.4, 75.8, 74.4, 73.4, 70.2, 63.5 were assigned to a glucopyranosyl unit. In the ^1^H-NMR spectrum, the signals at δ_H_ 7.54 (1H, d, *J* = 16.2 Hz) and 6.39 (1H, d, *J* = 16.2 Hz) implied the presence of a conjugated double bond with *E* geometry as deduced from the coupling constants. The signals at δ_H_ 7.44 (1H, d, *J* = 1.8 Hz), 7.33 (1H, dd, *J* = 7.8, 1.8 Hz) and 6.85 (1H, d, *J* = 7.8 Hz) showed an aromatic ring ABX spin system and implied the presence of a 1,3,4-trisubstituted aromatic ring. The signals at δ_H_ 7.53 (2H, d, *J* = 9.0 Hz) and 6.79 (2H, d, *J* = 9.0 Hz) implied the presence of a 1,4-disubstituted aromatic ring [10]. A comparison of its spectrum with that of 8,3′,4′-trihydroxyflavone-7-*O*-β-d-glucopyranoside (**27**), showed that the obvious difference in **3** was an additional *p*-coumaroyl [13] (Figure S1). HMBC correlation from H-6′′ (δ_H_ 4.44, 4.21) to C-9′′′ (δ_C_ 166.7) suggested the *p*-coumaroyl group was linked at C-6′′. Accordingly, compound **3** was elucidated as 8,3′,4′-trihydroxyflavone-7-*O*-(6′′-*O*-*p*-coumaroyl)-β-d-glucopyranoside (Figure 3).

In addition to three new glucosides **1**–**3**, fifty-three known compounds, identified as butylconiferin (**4**) [14], 2-methoxy-4-(2-propenyl)-phenyl-β-d-glucopyranoside (**5**) [15], 2-methoxy-4-(2′-hydroxy-ethyl)-phenol-1-*O*-β-d-glucopyranoside (**6**) [16], (1′*R*,2′*R*)-guaiacyl glycerol 3′-*O*-β-d-glucopyranoside (**7**) [17], *threo*-5-hydroxy-3,7-dimethoxyphenylpropane-8,9-diol (**8**) [18], 3-(4-hydroxy-3-methoxy-phenyl)-3-methoxypropane-1,2-diol (**9**) [19], 3-(4-hydroxy-3-methoxyphenyl)-propane-1,2-diol (**10**) [20], guaiacylglycerol (**11**) [21], ananasate (**12**) [22], *p*-hydroxyphenyl-6-*O*-trans-caffeoyl-β-d-alloside (1**3**) [23], 6′-*O*-caffeoyl-*p*-hydroxyacetophenone-4-*O*-β-d-glucopyranoside (**14**) [24], wilfordiol B (**15**) [25], 4,5-di-*O*-caffeoylquinic acid 1-methyl ether (**16**) [26], caffeoylcalleryanin (**17**) [27], 1-*O*-(*E*)-caffeoyl-β-d-gentiobiose (**18**) [28], plantasioside (**19**) [29], okanin-4′-*O*-(6′′-*O*-*p*-coumaroyl-β-d-glucopyranoside) (**20**) [8], (-)-4′-methoxy-7-*O*-(6′′-acetyl)-β-d-glucopyranosyl-8,3′-dihydroxy-flavanone (**21**), (−)-4′-methoxy-7-*O*-β-d-glucopyranosyl-8,3′-dihydroxyflavanone (**22**) [30], hesperetin-7-*O*-β-d-glucopyranoside (**23**) [31], apigenin (**24**) [32], 3′-hydroxyscutellarein-7-*O*-(6′′-*O*-proto-catechuoyl)-β-glucopyranoside (**25**) [33], quercetin-3-*O*-glucopyranoside (**26**) [34], 8,3′,4′-trihydroxyflavone-7-*O*-β-d-glucopyranoside (**27**) [13], luteolin-7-*O*-glucoside (**28**), 6-hydroxyluteolin-7-*O*-glucoside (**29**) [35], 3′′-(3-hydroxy-3-methylglutaroyl)-ester of 6-hydroxyluteolin-7-*O*-β-d-glucopyranoside (**30**) [36], luteolin-7-*O*-(β-d-glucopyranosyl)-2-glucopyranoside (**31**) [37], (*Z*)-7-*O*-β-d-glucopyranosyl-6,7,3′,4′-tetrahydroxyaurone (**32**) [38], sulfuretin-6-*O*-β-d-glucoside (**33**), maritimetin-6-*O*-β-d-glucoside (**34**) [39], dihydrophaseic acid (**35**) [40], caffeic acid (**36**), protocatechuic acid (**38**) [41], isoferuloyl ethylester (**37**) [42], 1,3,5-trimethoxybenzene (**39**) [43], vanillin (**40**) [44], 7*R*,11*R*-phytol (**41**) [45], 1-octacosanol (**42**) [46], indole-3-carboxylic acid (**43**) [47], 1*H*-indole-3-carboxadehyde (**44**) [48], niacinamide (**45**) [49], β-sitosterol (**46**) [50], stigmasterol (**47**) [51], hiziprafuran (**48**) [52], 5-hydroxy-2-furaldehyde (**49**) [53], α-tocopherol (**50**) [54], 4-hydroxy-2-furaldehyde (**51**) [55], α-tocospiro A (**52**) [56], ethyl linoleate (**53**), methyl linolenate (**54**) [57], tripalmitolein (**55**) [58], trilinolenin (**56**) [59] were isolated from *B.* *frondosa*. These compounds were identified by spectral analysis, and we found their spectral data were consistent with spectroscopic data reported in the corresponding literature.

### 2.2. Anti-Inflammatory Activity 

**Figure 4:**
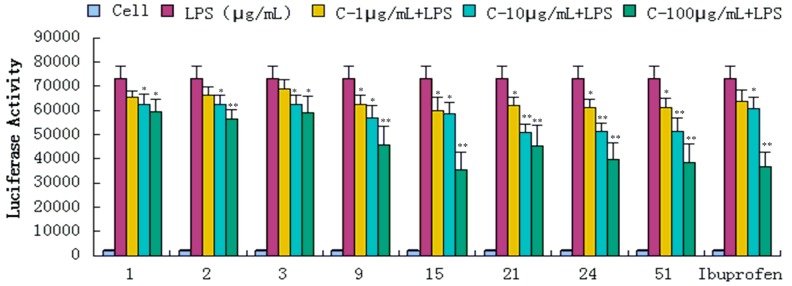
Inhibitory effects of compounds **1**, **2**, **3**, **9**, **15**, **21**, **24** and **51** (1, 10, 100 μg/mL) on NF-κB in luciferase activity assay. Data are expressed as mean ± S.E.M. of three independent experiments. Cell: cultures were not exposed to lipopolysaccharide (LPS); LPS: cultures were subjected to LPS; LPS + Drug: Compounds were added to the cultures during LPS; Positive control: LPS + Ibuprofen. * *p* < 0.05 *vs.* LPS; ** *p* < 0.01 *vs.* LPS.

**Figure 5:**
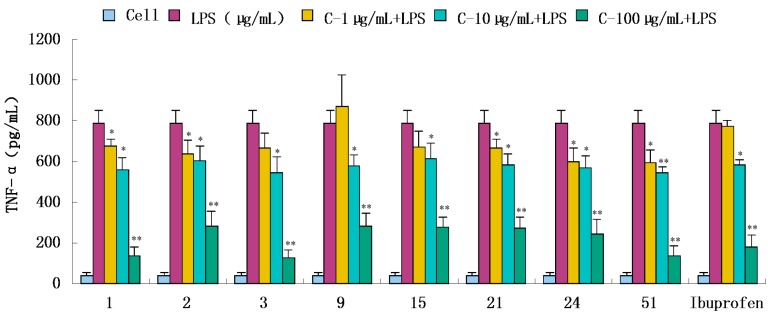
Inhibitory effects of compounds **1**, **2**, **3**, **9**, **15**, **21**, **24** and **51** (1, 10, 100 μg/mL) on TNF-α production stimulated by LPS (10 μg/mL) in RAW 264.7 cells (mouse leukemic monocyte macrophage cell line). Data are expressed as mean ± S.E.M. of three independent experiments. Cell: cultures were not exposed to lipopolysaccharide (LPS); LPS: cultures were subjected to LPS; LPS + Drug: Compounds were added to the cultures during LPS; Positive control: LPS + Ibuprofen. * *p* < 0.05 *vs.* LPS; ** *p* < 0.01 *vs.* LPS.

**Figure 6:**
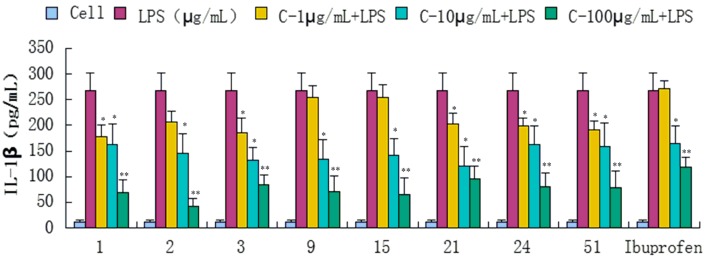
Inhibitory effects of compounds **1**, **2**, **3**, **9**, **15**, **21**, **24** and **51** (1, 10, 100 μg/mL) on IL-1β production stimulated by LPS (10 μg/mL) in RAW 264.7 cells (mouse leukemic monocyte macrophage cell line). Data are expressed as mean ± S.E.M. of three independent experiments. Cell: cultures were not exposed to lipopolysaccharide (LPS); LPS: cultures were subjected to LPS; LPS + Drug: Compounds were added to the cultures during LPS; Positive control: LPS + Ibuprofen. * *p* < 0.05 *vs.* LPS; ** *p* < 0.01 *vs.* LPS.

**Figure 7:**
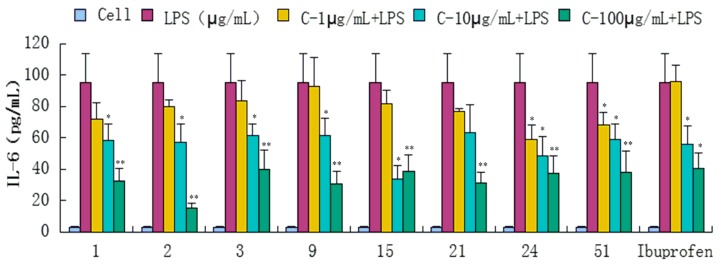
Inhibitory effects of compounds **1**, **2**, **3**, **9**, **15**, **21**, **24** and **51** (1, 10, 100 μg/mL) on IL-6 production stimulated by LPS (10 μg/mL) in RAW 264.7 cells (mouse leukemic monocyte macrophage cell line). Data are expressed as mean ± S.E.M. of three independent experiments. Cell: cultures were not exposed to lipopolysaccharide (LPS); LPS: cultures were subjected to LPS; LPS + Drug: Compounds were added to the cultures during LPS; Positive control: LPS + Ibuprofen. * *p* < 0.05 *vs.* LPS; ** *p* < 0.01 *vs.* LPS.

**Figure 8:**
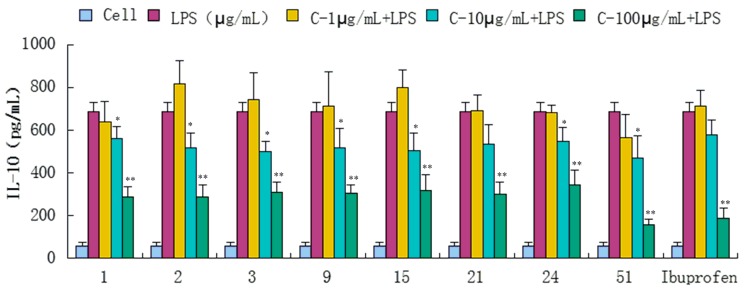
Inhibitory effects of compounds **1**, **2**, **3**, **9**, **15**, **21**, **24** and **51** (1, 10, 100 μg/mL) on IL-10 production stimulated by LPS (10 μg/mL) in RAW 264.7 cells (mouse leukemic monocyte macrophage cell line). Data are expressed as mean ± S.E.M. of three independent experiments. Cell: cultures were not exposed to lipopolysaccharide (LPS); LPS: cultures were subjected to LPS; LPS + Drug: Compounds were added to the cultures during LPS; Positive control: LPS + Ibuprofen. * *p* < 0.05 *vs.* LPS; ** *p* < 0.01 *vs.* LPS.

Compounds **1**–**34**, **36**, **39**, **43**, **47**, **51**, and **52** were evaluated for their anti-inflammatory activities in a luciferase assay (Table S1). Compared with the cell group, the luciferase activity of the LPS group was significantly enhanced, which indicated that the inflammatory cell model induced by LPS was constructed successfully. Then we have found the luciferase activity of compounds (**1**, **2**, **3**, **9**, **15**, **21**, **24** and **51**) group were significantly decreased by comparing with the LPS group, which showed that they had significant inhibitory effect on NF-κB activity. In addition, the luciferase activity decreased with the increase of sample concentration, which indicated that the inhibition of NF-κB activity was dose-dependent. In conclusion, compounds **1**, **2**, **3**, **9**, **15**, **21**, **24** and **51** showed significant inhibitory effect on NF-κB in 293-NF-κB-luciferase report cell line induced by LPS (Figure 4). The effects of compounds **1**, **2**, **3**, **9**, **15**, **21**, **24** and **51** on the inflammatory response were investigated further. The anti-inflammatory effects were evaluated by investigating the inhibitory activity of the compounds on the production of TNF-α, IL-1β, IL-6, and IL-10 in RAW 264.7 macrophages induced by LPS. For all assays, ibuprofen was used as a positive control. We have found the content of inflammatory cytokines TNF-α, IL-6, IL-1, IL-10 of the LPS group were significantly increased by comparing with the experimental results of the Cell group, which indicated that the monocyte RAW264.7 induced by LPS was constructed successfully. Compared with the LPS group, compounds **1**, **2**, **3**, **9**, **15**, **21**, **24** and **51** exhibited significant inhibitory activity on the production of above inflammation factors tested *in vitro* at concentrations of 1, 10 and 100 μg/mL, and the inhibition activity was dose-dependent (Figure 5, Figure 6, Figure 7 and Figure 8).

## 3. Experimental Section 

### 3.1. General Procedures

Optical rotations were measured on a Perkin-Elmer 341 Polarimeter (Perkin Elmer, Fremont, CA, USA). IR analyses were performed with a NEXUS 470 FT-IR spectrometer (Thermo-Nicolet, Madison, WI, USA). UV spectra were recorded on Shimadzu UV/VIS-240 recording spectrophotometer (Shimadzu, Tokyo, Japan). 1D- and 2D-NMR spectra were obtained on a Bruker Avance 600 NMR spectrometer (Bruker, Karlsruhe, Germany). HR-ESIMS were acquired on an Agilent 6220 TOF LC-MS instrument (Agilent, Santa Clara, CA, USA). Column chromatography was performed by using silica gel (100–200 and 200–300 mesh; Yantai Jiangyou Silica Gel Development Co. Ltd., Yantai, China), ODS (50 μm; YMC, Wilmington, NC, USA), MCI GEL (75–150 μm; Mitsubishi Chemical Corporation, Tokyo, Japan), Sephadex LH-20 (40–70 μm; Pharmacia Company, Uppsala, Sweden). Semi-preparative HPLC isolation was achieved with an Agilent 1200 instrument (Agilent, Santa Clara, CA, USA) equipped with a refractive index detector (RID), using a C18 column (250 mm × 10 mm × 5 μm, YMC) and eluting with MeOH–H_2_O at 2.0 mL/min. Precoated silica gel GF_254_ and HF_254_ plates were used for TLC, and Zones were visualized under UV light (254 nm and 365 nm) or by spraying with 10% H_2_SO_4_–EtOH followed by heating.

### 3.2. Plant Material

The plant of *B. Frondosa* was collected from Jiujiang, Jiangxi Province, during July 2013, and identified by Ceming Tan (Jiujiang Forest Herbarium, Jiangxi, China). A voucher specimen (No. 20130729) was deposited at the Department of Pharmacognosy of the Second Military Medical University.

### 3.3. Extraction and Isolation

The air-dried aerial parts of *B. frondosa* (10 kg) were extracted three times with 80% EtOH (100 L) under reflux. After removal of the solvent by evaporation under vacuum, the residue was suspended in water (10 L) and then successively partitioned with petroleum ether, EtOAc and *n*-BuOH (3 × 15 L), respectively. 

The petroleum ether-soluble part (279 g) was fractionated by silica gel CC and and eluted with petroleum ether–ethyl acetate in increasing polarity (100:1→10:1) to yield six fractions (Fr.1–Fr.6). Fr.1 (42 g) was subjected to repeated silica gel CC using a step gradient of petroleum ether–EtOAc (80:1→60:1→40:1→20:1→10:1) and further purified by Sephadex LH-20 column eluted with CH_2_Cl_2_–MeOH (1:1) to afford compounds **55** (85 mg). Fr.2 (50 g) was chromatographed on silica gel column and eluted with increasing polarities of petroleum ether–EtOAc (60:1→30:1→10:1) to give four subfractions (Fr.2-1–Fr.2-4). Fr.2-1 (5 g) was chromatographed on silica gel CC using a step gradient of petroleum ether–EtOAc (50:1→20:1→10:1) and finally purified by Sephadex LH-20 column eluted with CH_2_Cl_2_–MeOH (1:1) to yield compound **53** (196 mg). Fr.2-2 (1.4 g) and Fr.2-4 (2 g) was respectively separated by Sephadex LH-20 column eluted with CH_2_Cl_2_–MeOH (1:1) to yield compound **56** (500 mg). Fr.3 (43 g) was subjected to repeated silica gel CC using a step gradient of petroleum ether–EtOAc (40:1→30:1→20:1→10:1→5:1) and further purified by Sephadex LH-20 column eluted with CH_2_Cl_2_–MeOH (1:1) to yield compounds **54** (450 mg). Fr.4 (40 g) was subjected to repeated silica gel CC using a step gradient of petroleum ether–EtOAc (30:1→20:1→10:1→5:1→1:1) and further purified by Sephadex LH-20 column eluted with CH_2_Cl_2_–MeOH (1:1) to yield compounds **42** (2 mg). Fr.5 (34 g) was subjected to repeated Silica gel CC using a step gradient of petroleum ether–EtOAc (20:1→10:1→5:1→1:1) and further purified by Sephadex LH-20 column eluted with CH_2_Cl_2_–MeOH (1:1) to afford compounds **41** (27 mg).

The EtOAc-soluble part (214 g) was fractionated by silica gel CC eluted with CH_2_Cl_2_–MeOH (50:1→4:1) to give eight fractions (Fr.7–Fr.14). Fr.7 (30 g) was subjected to repeated silica gel CC eluted with CH_2_Cl_2_-MeOH (40:1→20:1→10:1→4:1) and further purified by Sephadex LH-20 column eluted with CH_2_Cl_2_–MeOH (1:1) to afford compounds **47** (8 mg), **50** (3 mg) and **52** (10 mg). Fr.8 (29 g) was subjected to silica gel CC eluted with CH_2_Cl_2_–MeOH (30:1→20:1→10:1→4:1), further separated by ODS CC eluted with MeOH–H_2_O (40%→60%→80%) and finally purified by Sephadex LH-20 column eluted with CH_2_Cl_2_–MeOH (1:1) to afford compounds **46** (100 mg), **48** (23 mg) and **49** (19 mg). Fr.9 (23 g) was subjected to silica gel CC eluted with CH_2_Cl_2_–MeOH (20:1→10:1→4:1), further separated by ODS CC eluted with MeOH–H_2_O (40%→60%→80%) and finally purified by Sephadex LH-20 column eluted with CH_2_Cl_2_–MeOH (1:1) to afford compounds **43** (12 mg) and **44** (3 mg). Fr.10 (25 g) was subjected to silica gel CC eluted with CH_2_Cl_2_–MeOH (20:1→10:1→4:1→1:1) and finally purified by Sephadex LH-20 column eluted with CH_2_Cl_2_–MeOH (1:1) to afford compounds **45** (10 mg) and **51** (4 mg). Fr.11 (24 g) was subjected to ODS CC eluted with MeOH–H_2_O (40%→60%→80%) and finally purified by Sephadex LH-20 column eluted with CH_2_Cl_2_–MeOH (1:1) to afford compound **40** (17 mg). Fr.12 (15 g) was subjected to repeated ODS CC eluted with MeOH–H_2_O (40%→60%→80%) and finally purified by Sephadex LH-20 column eluted with CH_2_Cl_2_–MeOH (1:1) to afford compounds **35** (14 mg) and **39** (6 mg). Fr.13 (23 g) was subjected to ODS CC eluted with MeOH–H_2_O (40%→60%→80%) , further separated by silica gel CC eluted with CH_2_Cl_2_-MeOH (10:1→4:1→1:1) and finally purified by Sephadex LH-20 column eluted with CH_2_Cl_2_–MeOH (1:1) to afford compounds **36** (74 mg) and **38** (574 mg). Fr.14 (17 g) was subjected to ODS CC eluted with MeOH–H_2_O (50%→70%→90%) and finally purified by Sephadex LH-20 column eluted with CH_2_Cl_2_–MeOH (1:1) to afford compound **37** (3 mg). 

The *n*-BuOH-soluble part (325 g) was fractionated by silica gel CC eluted with CH_2_Cl_2_–MeOH (20:1→2:1) to give five fractions (Fr.15–Fr.19). Fr.15 (45 g) was subjected to ODS column with a step gradient elution (50%→80%, MeOH in H_2_O) to afford two subfractions (Fr.15-1–Fr.15-2). Fr.15-1 (15 g) was purified by semi-preparative HPLC (30% MeOH–H_2_O, 2 mL/min) to give compounds **2** (2 mg), **4** (5 mg), **7** (2 mg) and **13** (3 mg). Fr.15-2 (20 g) was purified by Sephadex LH-20 column eluted with CH_2_Cl_2_–MeOH (1:1) to afford compounds **5** (595 mg), **6** (5 mg) and **28** (12 mg). Fr.16 (60 g) was subjected to MCI column with a step gradient elution (50%→70%→90%, MeOH in H_2_O) to afford three subfractions (Fr.16-1–Fr.16-3). Fr.16-1 (18 g) was purified by semi-preparative HPLC (25% MeOH–H_2_O, 2 mL/min) to give compounds **8** (13 mg), **9** (2 mg) and **10** (4 mg). Fr.16-2 (20 g) was purified by Sephadex LH-20 column eluted with CH_2_Cl_2_–MeOH (1:1) to give compounds **11** (5 mg) and 1**2** (7 mg). Fr.16-3 (10 g) was purified by semi-preparative HPLC (40% MeOH–H_2_O, 2 mL/min) to give compounds **1** **(**4 mg) and **33** **(**4 mg). Fr.17 (65 g) was subjected to ODS column with a step gradient elution (5%–20%–50%–70%–90%, MeOH in H_2_O) to afford two subfractions (Fr.17-1–Fr.17-2). Fr.17-1 (28 g) was purified by semi-preparative HPLC (25% MeOH–H_2_O, 2 mL/min) to give compounds **18** (20 mg), **19** (20 mg), **21** (23 mg) and **22** (7 mg). Fr.17-2 (29 g) was purified by semi-preparative HPLC (35% MeOH–H_2_O, 2 mL/min) to give compounds **14** **(**8 mg) and **20** (6 mg). Fr.18 (58 g) was subjected to MCI GEL column with a step gradient elution (5%–20%–50%–70%–90%, MeOH in H_2_O) to afford two subfractions (Fr.18-1–Fr.18-2). Fr.18-1 (21 g) was purified by semi-preparative HPLC (30% MeOH–H_2_O, 2 mL/min) to give compounds **23** (4 mg), **26** (5 mg), **32** (20 mg) and **34** (32 mg). Fr.18-2 (25 g) was purified by semi-preparative HPLC (60% MeOH–H_2_O, 2 mL/min) to give compounds **15** (57 mg), **16** (8 mg) and **24** (3 mg). Fr.19 (58 g) was subjected to ODS column with a step gradient elution (5%–20%–50%–70%–100%, MeOH in H_2_O) to afford two subfractions (Fr.19-1–Fr.19-2). Fr.19-1 (22 g) was purified by semi-preparative HPLC (30% MeOH–H_2_O, 2 mL/min) to give compounds **3** (15 mg), **27** (14 mg), **29** (5 mg) and **30** (5 mg). Fr.19-2 (21 g) was purified by semi-preparative HPLC (65% MeOH-H_2_O, 2 mL/min) to give compounds **17** (11 mg), **25** (15 mg) and **31** (11 mg). 

### 3.4. Luciferase Assay 

The NF-κB 293 cells were cultured in a DMEM medium supplemented with 10% fetal bovine serum (FBS). The cells were pretreated with these forty compounds at concentrations of 1, 10 and 100 μg/mL for 4 h and then stimulated with 10 μg/mL lipopolysaccharide (LPS) for 24 h. The cells were rinsed twice with phosphate-buffered saline (PBS, pH 7.4) and lysed with passive lysis buffer (Promega, Madison, WI, USA). Then inhibitory effect on NF-κB was analyzed using the luciferase assay system (Promega) according to the manufacturer′s instructions [60].

### 3.5. Measurement of TNF-α, IL-1β, IL-6, and IL-10

The cells were cultured in serum-free medium for 8 h and then incubated in medium containing 1, 10 and 100 μg/mL of compounds **1**, **2**, **3**, **9**, **15**, **21**, **24** and **51** for 2 h. The cells were then treated with 10 μg/mL of LPS for 24 h. Ibuprofen (1, 10 and 100 μg/mL) was used as a positive control. The supernatants of cell culture were harvested and centrifuged at 3000× *g* at 4 °C for 2 min for the analysis of TNF-α, IL-1β, IL-6, and IL-10. Enzyme-linked immunosorbent assays for detecting the cytokines in the supernatants were carried out according to the instructions provided by the manufacturer. Finally, the standard provided with the kits was used to quantify each cytokine in the supernatants [61].

### 3.6. Characterization of Compounds

*(3E,5E,11E)-Tridecatriene-7,9-diyne-1,2,13-triol-2-O-β-d-glucopyranoside* (**1**): brownish amorphous powder; ${\text{[α]}}_{D}^{20}$ −16.25 (*c* 0.04, MeOH); UV (MeOH) *λ*_max_ (log ɛ) 339, 317, 298, 268, 253 nm; IR (KBr) *v*_max_ 3362, 3005, 2922, 2852, 2025, 1658, 1632, 1467, 1423, 1411, 1383, 1075, 812 cm^−1^; ^1^H-NMR (DMSO-*d*_6_, 600 MHz): δ 6.81 (1H, dd, *J* = 15.6, 10.8 Hz, H-5), 6.48 (1H, ddd, *J* = 16.2, 9.0, 8.4 Hz, H-12), 6.41 (1H, dd, *J* = 15.6, 10.8 Hz, H-4), 6.00 (1H, dd, *J* = 15.6, 5.4 Hz, H-3), 5.88 (1H, d, *J* = 15.6 Hz, H-6), 5.86 (1H, d, *J* = 16.2Hz, H-11), 4.27 (1H, d, *J* = 7.8 Hz, H-1′), 4.22 (1H, d, *J* = 4.8 Hz, H-2), 4.05 (2H, dd, *J* = 4.2, 2.4 Hz, H-13), 3.62 (1H, d, *J* = 10.2 Hz, H-6′*α*), 3.45 (2H, m, H-1), 3.43 (1H, m, H-6′β), 3.14 (1H, m, H-5′), 3.05 (1H, m, H-3′), 3.04 (1H, m, H-4′), 2.97 (1H, m, H-2′). ^13^C-NMR (DMSO-*d*_6_, 150 MHz) : δ 63.7 (C-1), 79.9 (C-2), 137.9 (C-3), 129.8 (C-4), 145.5 (C-5), 109.1 (C-6), 81.5 (C-7), 76.2 (C-8), 81.9 (C-9), 73.8 (C-10), 106.4 (C-11), 149.5 (C-12), 61.2 (C-13), 102.7 (C-1′), 74.0 (C-2′), 77.1 (C-3′), 70.2 (C-4′), 76.8 (C-5′), 61.0 (C-6′). HR-ESIMS: *m*/*z* 403.1371 [M + Na]^+^ (calcd. for C_19_H_24_O_8_Na, 403.1369).

*2′-Butoxyethylconiferin* (**2**): white amorphous powder; ${\text{[α]}}_{D}^{20}$ −27.22 (*c* 0.06, MeOH); UV(MeOH) *λ*_max_ (log ɛ) 259, 202 nm; IR (KBr) *ν*_max_ 3438, 2922, 2853, 2026, 1725, 1710, 1630, 1512, 1462, 13834, 1127 cm^−1^; ^1^H-NMR (CD_3_OD, 600 MHz): δ 7.06 (1H, d, *J* = 8.4 Hz, H-5), 7.03 (1H, d, *J* = 1.8 Hz, H-2), 6.90 (1H, dd, *J* = 8.4, 1.8 Hz, H-6), 6.54 (1H, d, *J* = 15.6 Hz, H-7), 6.20 (1H, dd, *J* = 15.6, 6.0 Hz, H-8), 4.83 (1H, overlap, H-1′′′), 4.12 (2H, dd, *J* = 6.0, 1.2 Hz, H-9), 3.83 (3H, s, OCH_3_), 3.84 (1H, m, H-6′′′α), 3.65 (1H, m, H-6′′′β), 3.58 (2H, m, H-1′), 3.56 (2H, m, H-2′), 3.46 (1H, m, H-4′′′), 3.45 (1H, m, H-2′′′), 3.44 (2H, m, H-1′′), 3.36 (1H, m, H-3′′′), 3.35 (1H, m, H-5′′′), 1.52 (2H, m, H-2′′), 1.34 (2H, m, H-3′′), 0.88 (3H, t, *J* = 7.2 Hz, H-4′′). ^13^C-NMR (CD_3_OD, 150MHz): δ 133.4 (C-1), 111.4 (C-2), 150.9 (C-3), 147.8 (C-4), 117.9 (C-5), 120.9 (C-6), 133.3 (C-7), 125.9 (C-8), 72.7 (C-9), 70.5 (C-1′), 71.2 (C-2′), 72.1 (C-1′′), 32.8 (C-2′′), 20.3 (C-3′′), 14.2 (C-4′′), 102.7 (C-1′′′), 74.9 (C-2′′′), 78.2 (C-3′′′), 71.3 (C-4′′′), 77.8 (C-5′′′), 62.5 (C-6′′′), 56.7 (OCH_3_). HR-ESIMS *m/z* 477.1917 [M + Cl]^−^ (calcd. for C_22_H_34_O_9_Cl, 477.1891). 

*8,3′,4′-Trihydroxyflavone-7-O-(6′′-O-p-coumaroyl)-β-d-glucopyranoside* (**3**): brownish-yellow amorphous powder; ${\text{[α]}}_{D}^{25}$ +7.22 (*c* 0.03, MeOH); UV (MeOH) *λ*_max_ (log ɛ) 414, 316 nm; IR (KBr) *v*_max_ 3405, 2923, 2853, 2026, 1688, 1649, 1603, 1513, 1443, 1383, 1278, 1168, 1129, 1076 cm^−1^; ^1^H-NMR (DMSO-*d*_6_, 600 MHz): δ 7.54 (1H, d, *J* = 16.2 Hz, H-7′′′), 7.53 (2H, d, *J* = 9.0 Hz, H-2′′′, 5′′′), 7.44 (1H, d, *J* = 1.8 Hz, H-2′), 7.33 (1H, dd, *J* = 7.8, 1.8 Hz, H-6′), 7.11 (1H, d, *J* = 8.4 Hz, H-5), 7.04 (1H, d, *J* = 8.4 Hz, H-6), 6.85 (1H, d, *J* = 7.8 Hz, H-5′), 6.79 (2H, d, *J* = 9.0 Hz, H-3′′′, 6′′′), 6.67 (1H, s, H-3), 6.39 (1H, d, *J* = 16.2 Hz, H-8′′′), 5.02 (1H, d, *J* = 7.8 Hz, H-1′′), 4.44 (1H, d, *J* = 9.6 Hz, H-6′′α), 4.21 (1H, dd, *J* = 12.0, 7.2 Hz, H-6′′β), 3.75 (1H, m, H-5′′), 3.43 (1H, m, H-2′′), 3.37 (1H, m, H-3′′), 3.27 (1H, m, H-4′′). ^13^C-NMR (DMSO-*d*_6_, 150 MHz) : δ 145.8 (C-2), 113.2 (C-3), 182.6 (C-4), 114.4 (C-5), 112.0 (C-6), 152.3 (C-7), 132.8 (C-8), 117.4 (C-9), 154.4 (C-10), 123.6 (C-1′), 118.6 (C-2′), 145.7 (C-3′), 148.7 (C-4′), 116.3 (C-5′), 125.2 (C-6′)), 101.4 (C-1′′), 73.4 (C-2′′), 75.8 (C-3′′), 70.2 (C-4′′), 74.4 (C-5′′), 63.5 (C-6′′), 125.3 (C-1′′′), 130.6 (C-2′′′), 116.1 (C-3′′′), 160.2 (C-4′′′), 116.1 (C-5′′′), 130.6 (C-6′′′), 145.2 (C-7′′′), 114.2 (C-8′′′), 166.7 (C-9′′′). HR-ESIMS: *m/z* 593.1316 [M − H]^−^ (calcd for C_30_H_2__5_O_13_, 593.1295). 

## 4. Conclusions

A new polyacetylene glucoside (3*E*,5*E*,11*E*)-tridecatriene-7,9-diyne-1,2,13-triol-2-*O*-β-d-glucopyranoside (**1**), a new phenylpropanoid glucoside, 2′-butoxyethylconiferin (**2**), and a new flavonoid glycoside, 8,3′,4′-trihydroxyflavone-7-*O*-(6′′-*O*-*p*-coumaroyl)-β-d-glucopyranoside (**3**), together with fifty-three known compounds **4**‒**56** have been obtained from *B. frondosa*. Except compounds **20**, **24**, **26**, **28**, **33**, **34**, the other compounds were reported to isolate from *B. frondosa* for the first time. Compounds **1**, **2**, **3**, **9**, **15**, **21**, **24** and **51** showed significant activities in anti-inflammatory assays. And they exhibited good anti-inflammatory effects in a dose-dependent manner. The observed potential anti-inflammatory activity warrants further investigations.

## Supplementary Materials

Supplementary data (Figure S1, 1D-, 2D-NMR spectra and HR-ESIMS data of compounds **1**, **2** and **3**, HPLC profiles of compounds **1**, **2**, **3**, **9**, **15**, **21**, **24**, **51** and EtOH extract of *B. Frondosa*, Table S1) associated with this article are available online.

Supplementary materials can be accessed at: http://www.mdpi.com/1420-3049/20/10/18496/s1.
