# Supplementary material for: Anti-Inflammatory Constituents from Bidens frondosa
Source: Molecules. 2015 Oct 9;20(10):18496–510. doi: 10.3390/molecules201018496 (PMC6332032; doi:10.3390/molecules201018496)
Supplement: Supplementary file 1 [file molecules-20-18496-s001.pdf]

# Supplementary Material

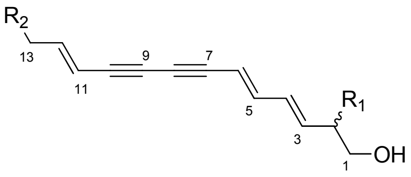

| | $R_1$ | $R_2$ |
|---|---|---|
| **1** | **O-β-glu** | **OH** |
| **1a** | **OH** | **O-β-glu** |

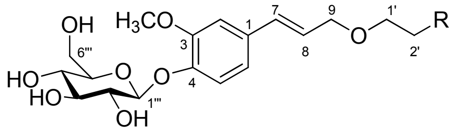

| | R |
|---|---|
| **2** | **$OCH_2CH_2CH_2CH_3$** |
| **2a** | **H** |

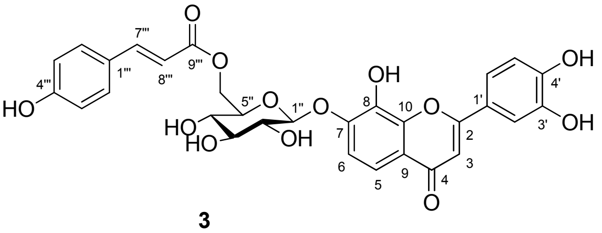

**3**

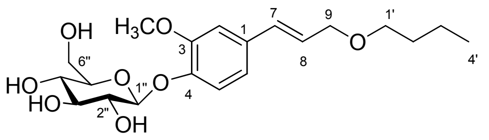

**4**

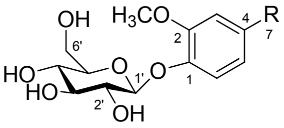

| | R |
|---|---|
| **5** | **$CH_2CHCH_2$** |
| **6** | **$CH_2CH_2OH$** |

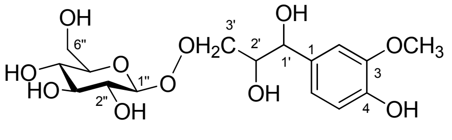

**7**

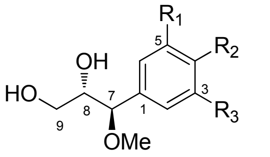

| | $R_1$ | $R_2$ | $R_3$ |
|---|---|---|---|
| **8** | **OH** | **H** | **OMe** |
| **9** | **OMe** | **OH** | **H** |

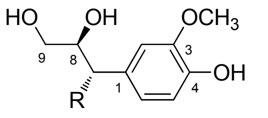

| | R |
|---|---|
| **10** | **H** |
| **11** | **OH** |

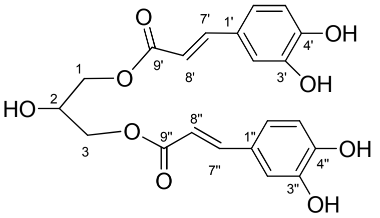

**12**

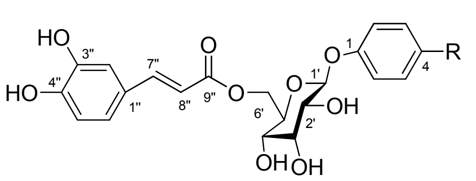

| | R |
|---|---|
| **13** | **OH** |
| **14** | **CH3CO** |

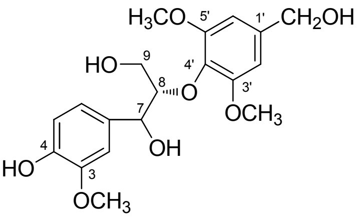

**15**

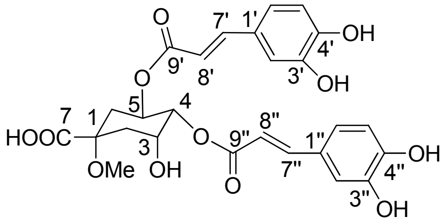

**16**

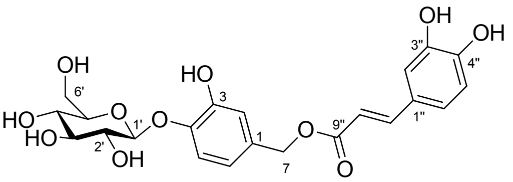

**17**

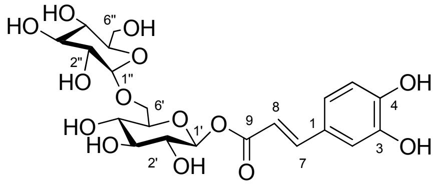

**18**

**Figure S1.** *Cont.*

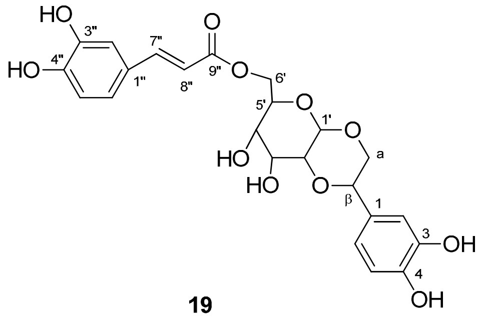

**19**

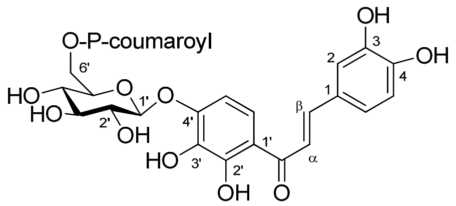

**20**

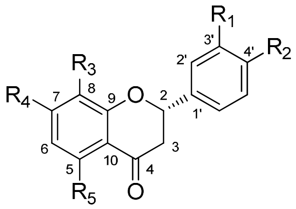

| | $R_1$ | $R_2$ | $R_3$ | $R_4$ | $R_5$ |
|---|---|---|---|---|---|
| **21** | **OH** | **OMe** | **OH** | **OGlu-6"-O-Ac** | **H** |
| **22** | **OH** | **OMe** | **OH** | **OGlu** | **H** |
| **23** | **OMe** | **OH** | **H** | **OGlu** | **OH** |

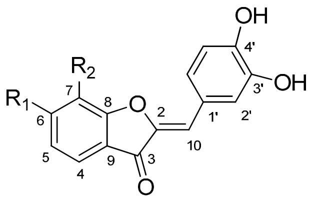

| | $R_1$ | $R_2$ |
|---|---|---|
| **32** | **OH** | **OGlu-6"-O-Ac** |
| **33** | **OGlu** | **H** |
| **34** | **OH** | **OGlu** |

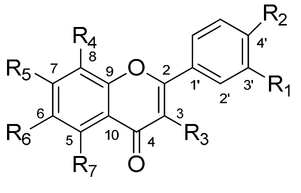

| | $R_1$ | $R_2$ | $R_3$ | $R_4$ | $R_5$ | $R_6$ | $R_7$ |
|---|---|---|---|---|---|---|---|
| **24** | **H** | **OH** | **H** | **H** | **OH** | **H** | **OH** |
| **25** | **OH** | **OH** | **H** | **H** | **OGlu-6"-O-protocatechuoyl** | **OH** | **OH** |
| **26** | **OH** | **OH** | **OGlu** | **H** | **OH** | **H** | **OH** |
| **27** | **OH** | **OH** | **H** | **OH** | **OGlu** | **H** | **H** |
| **28** | **OH** | **OH** | **H** | **H** | **OGlu** | **H** | **OH** |
| **29** | **OH** | **OH** | **H** | **H** | **OGlu** | **OH** | **OH** |
| **30** | **OH** | **OH** | **H** | **H** | **OGlu-3"-O-HMG-ester** | **OH** | **OH** |
| **31** | **OH** | **OH** | **H** | **H** | **OGlu-6"-O-Glu** | **H** | **OH** |

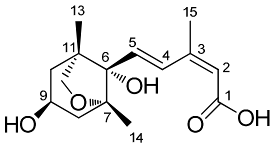

**35**

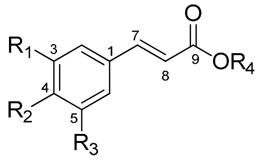

| | $R_1$ | $R_2$ | $R_3$ | $R_4$ |
|---|---|---|---|---|
| **36** | **OH** | **OH** | **H** | **H** |
| **37** | **OH** | **OMe** | **H** | **$CH_2CH_3$** |

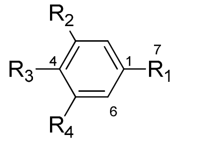

| | $R_1$ | $R_2$ | $R_3$ | $R_4$ |
|---|---|---|---|---|
| **38** | **COOH** | **OH** | **OH** | **H** |
| **39** | **OMe** | **OMe** | **H** | **OMe** |
| **40** | **CHO** | **H** | **OMe** | **OH** |

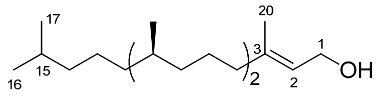

**41**

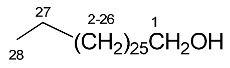

**42**

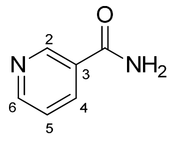

**45**

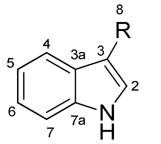

| | R |
|---|---|
| **43** | **COOH** |
| **44** | **CHO** |

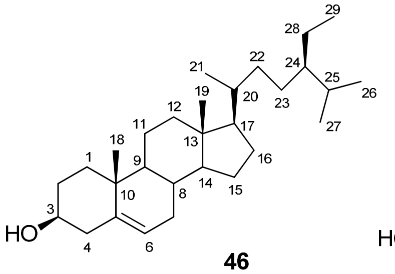

**46**

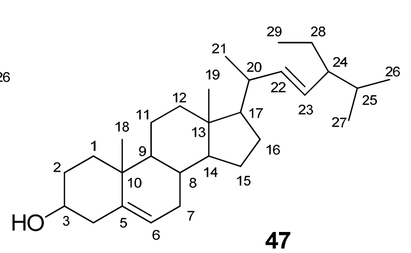

**47**

**Figure S1.** *Cont.*

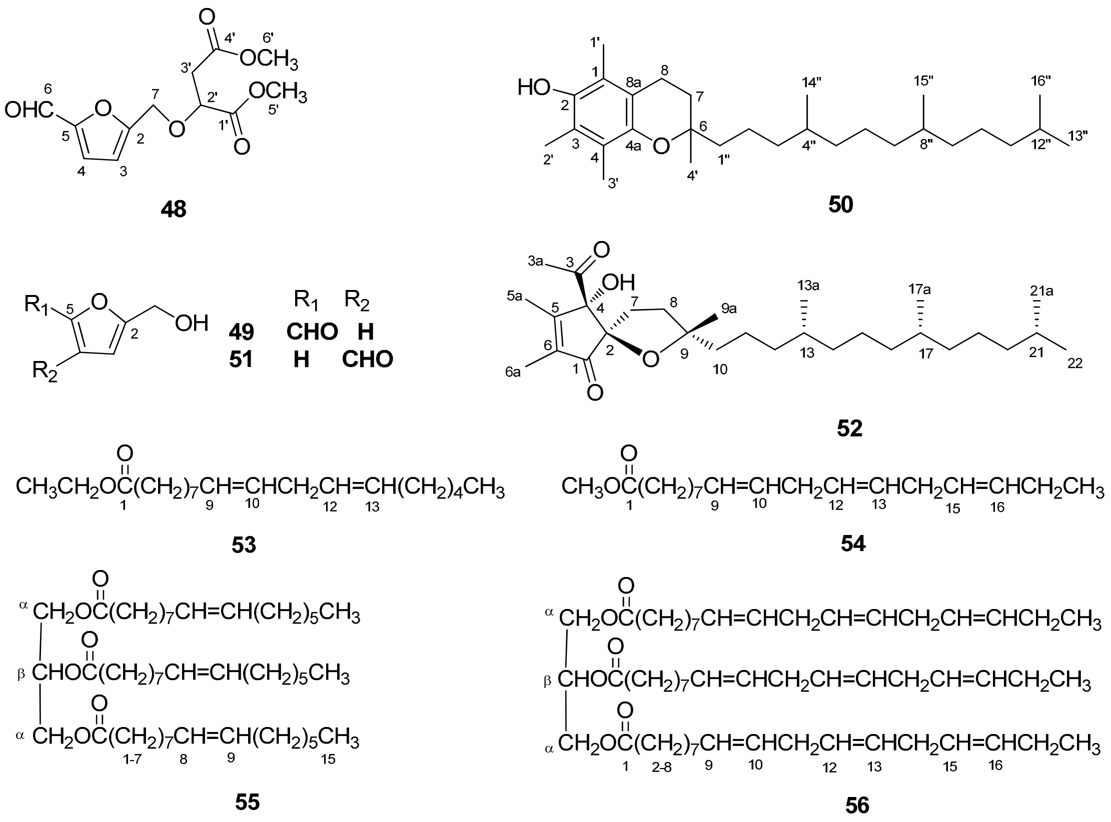


**Figure S1.** Structures of compounds **1**–**56**.

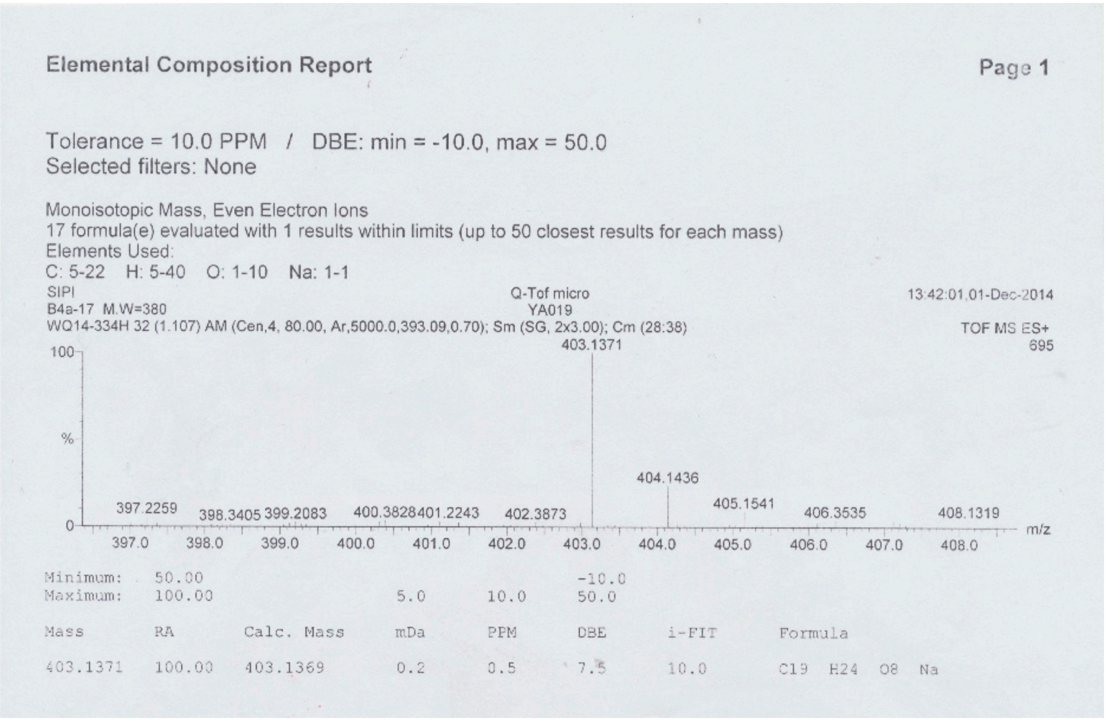

Elemental Composition Report

Page 1

Tolerance = 10.0 PPM / DBE: min = -10.0, max = 50.0
Selected filters: None

Monoisotopic Mass, Even Electron Ions
17 formula(e) evaluated with 1 results within limits (up to 50 closest results for each mass)
Elements Used:
C: 5-22 H: 5-40 O: 1-10 Na: 1-1

SIPI Q-Tof micro 13:42:01.01-Dec-2014
B4a-17 M.W=380 YA019
WQ14-334H 32 (1.107) AM (Cen,4, 80.00, Ar,5000.0,393.09,0.70); Sm (SG, 2x3.00); Cm (28:38) TOF MS ES+ 695



| | | | | | | | |
|---|---|---|---|---|---|---|---|
| Minimum: | 50.00 | | | | -10.0 | | |
| Maximum: | 100.00 | | 5.0 | 10.0 | 50.0 | | |

| Mass | RA | Calc. Mass | mDa | PPM | DBE | i-FIT | Formula |
|---|---|---|---|---|---|---|---|
| 403.1371 | 100.00 | 403.1369 | 0.2 | 0.5 | 7.5 | 10.0 | C19 H24 O8 Na |

**Figure S2.** HRESI mass spectrum of compound **1**.

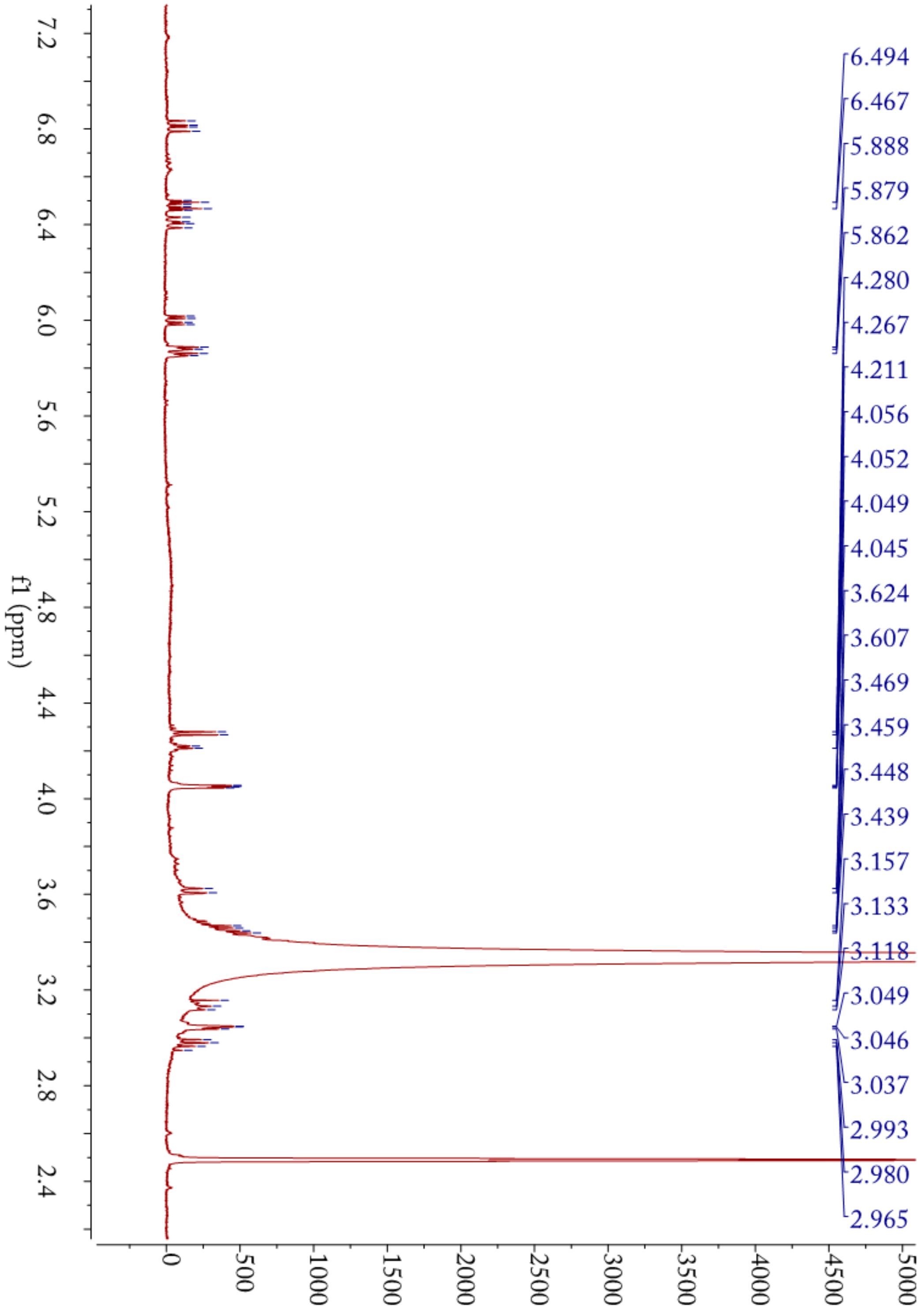


**Figure S3.** $^{1}$H-NMR spectrum of compound **1** (600 MHz, in DMSO-$d_6$).

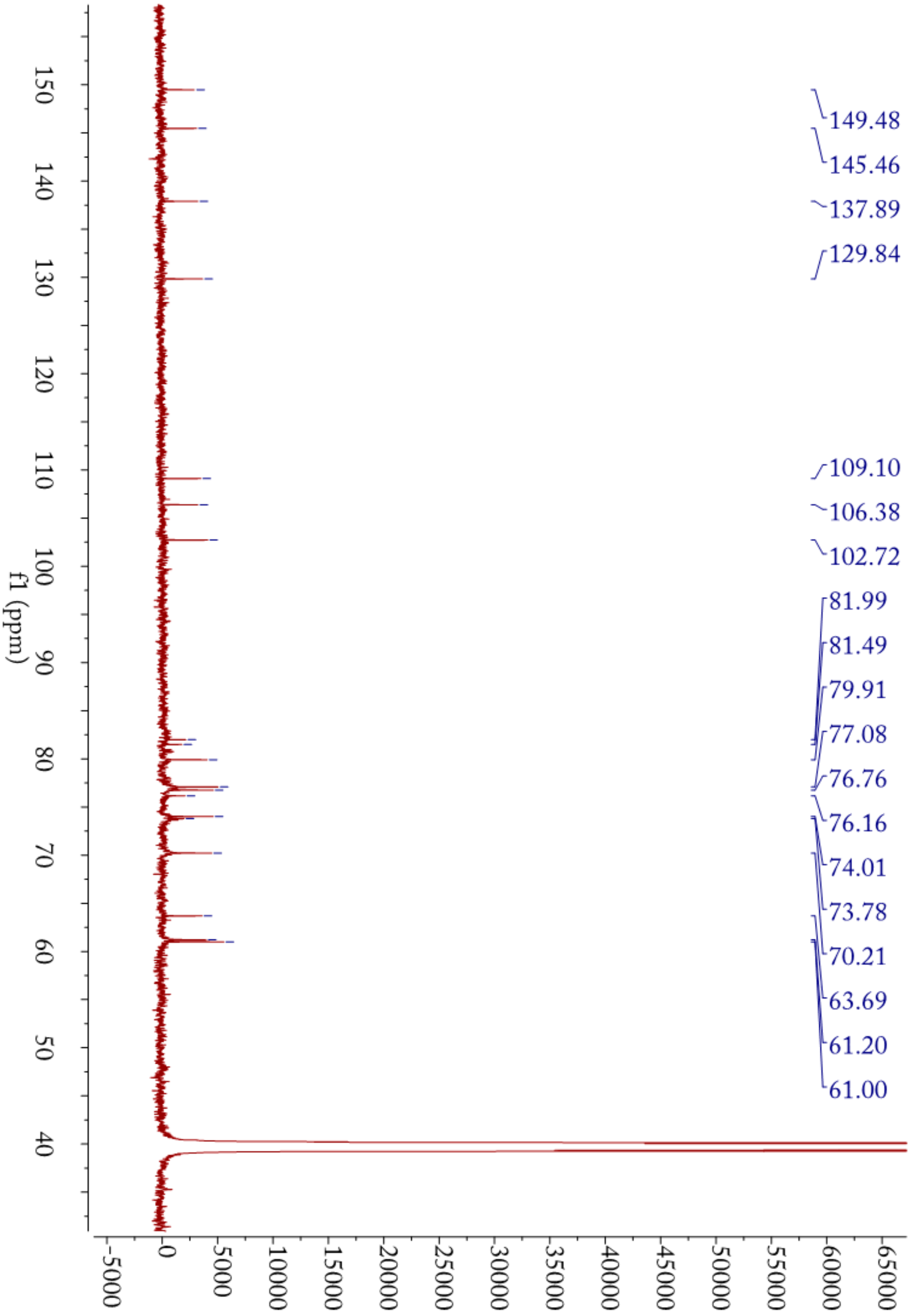


**Figure S4.** $^{13}$C-NMR spectrum of compound **1** (150 MHz, in DMSO-*d*$_6$).

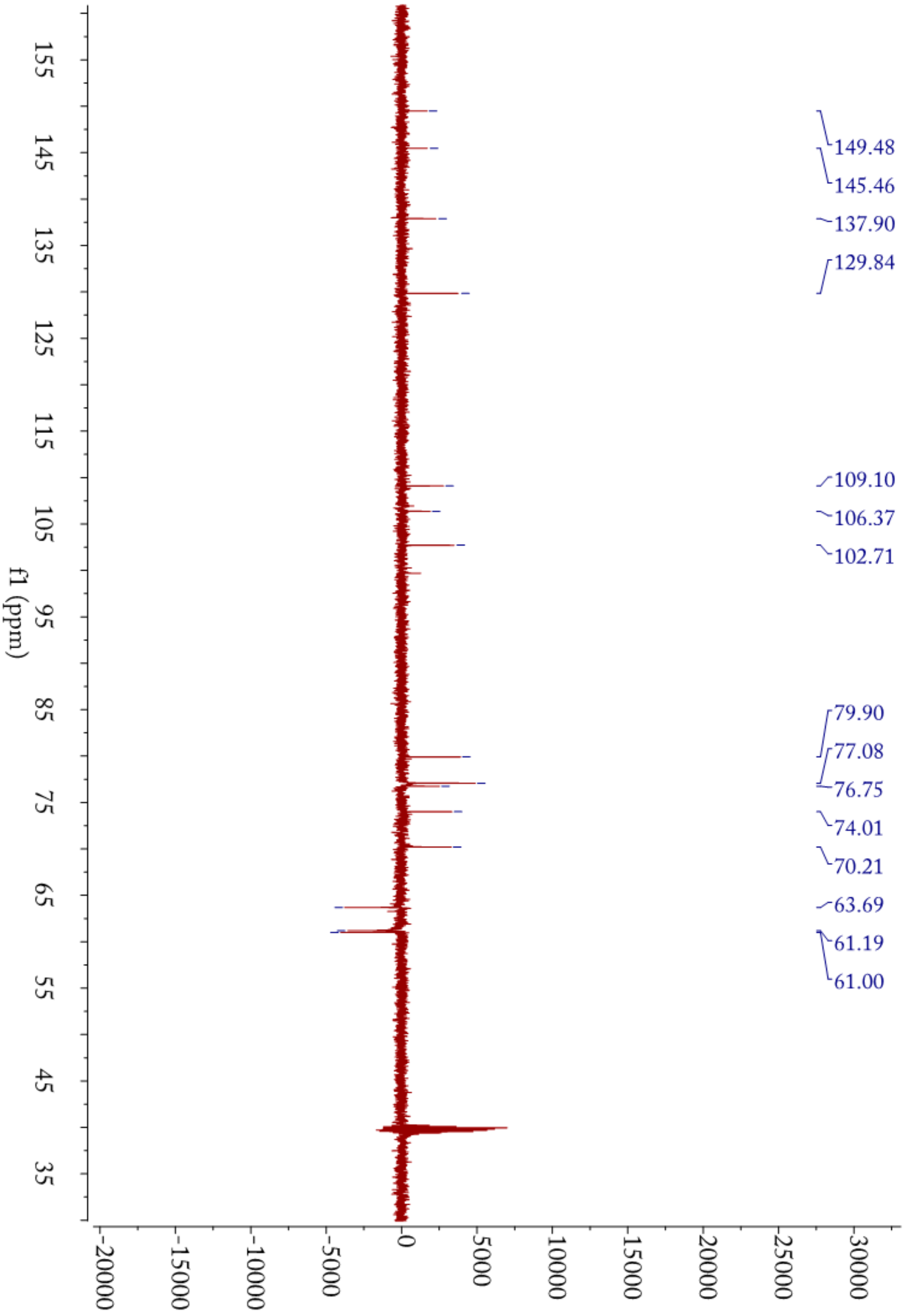


**Figure S5.** DEPT spectrum of compound **1** (150 MHz, in DMSO-$d_6$).

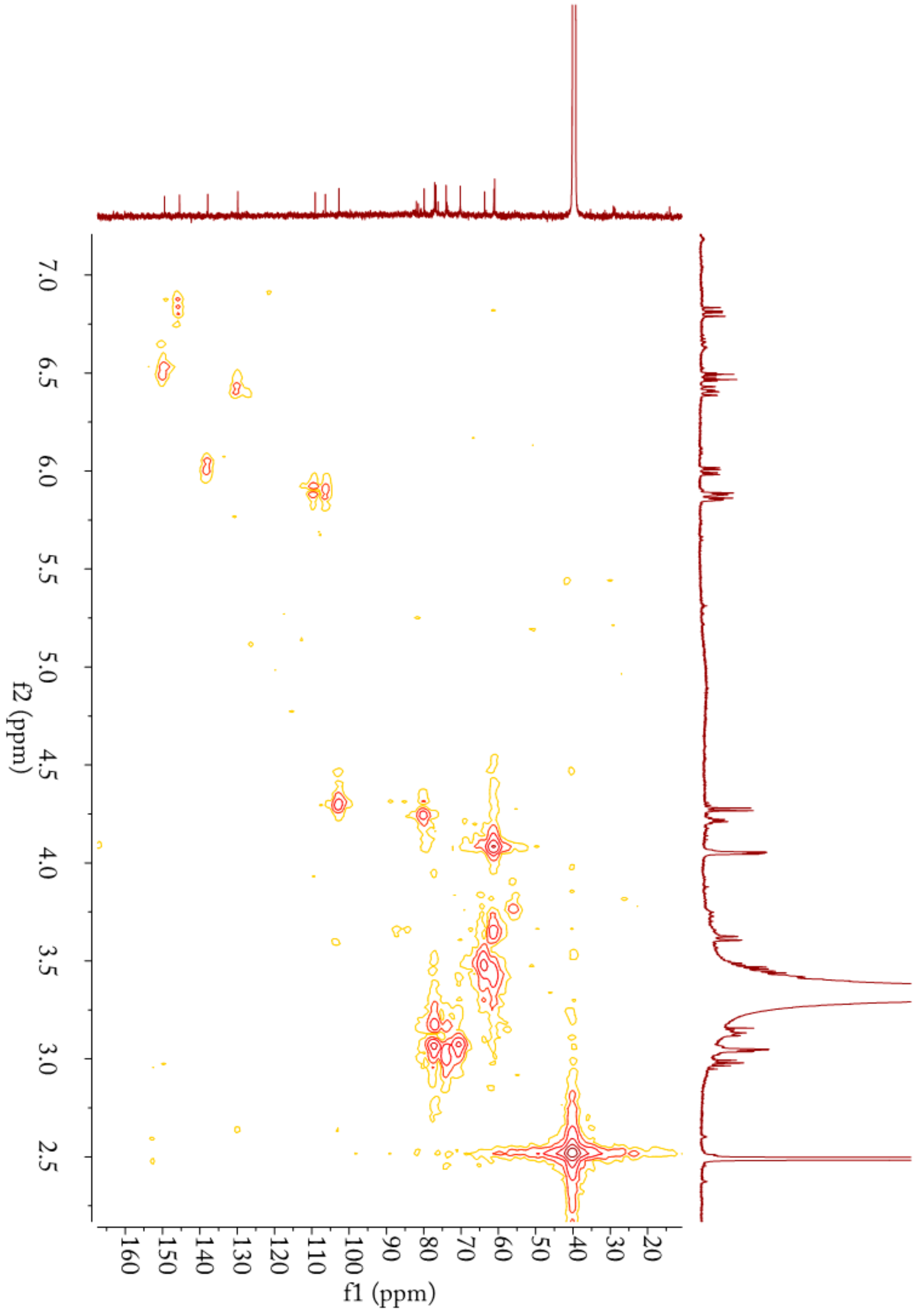


**Figure S6.** HSQC spectrum of compound **1** (600 MHz, in DMSO-$d_6$).

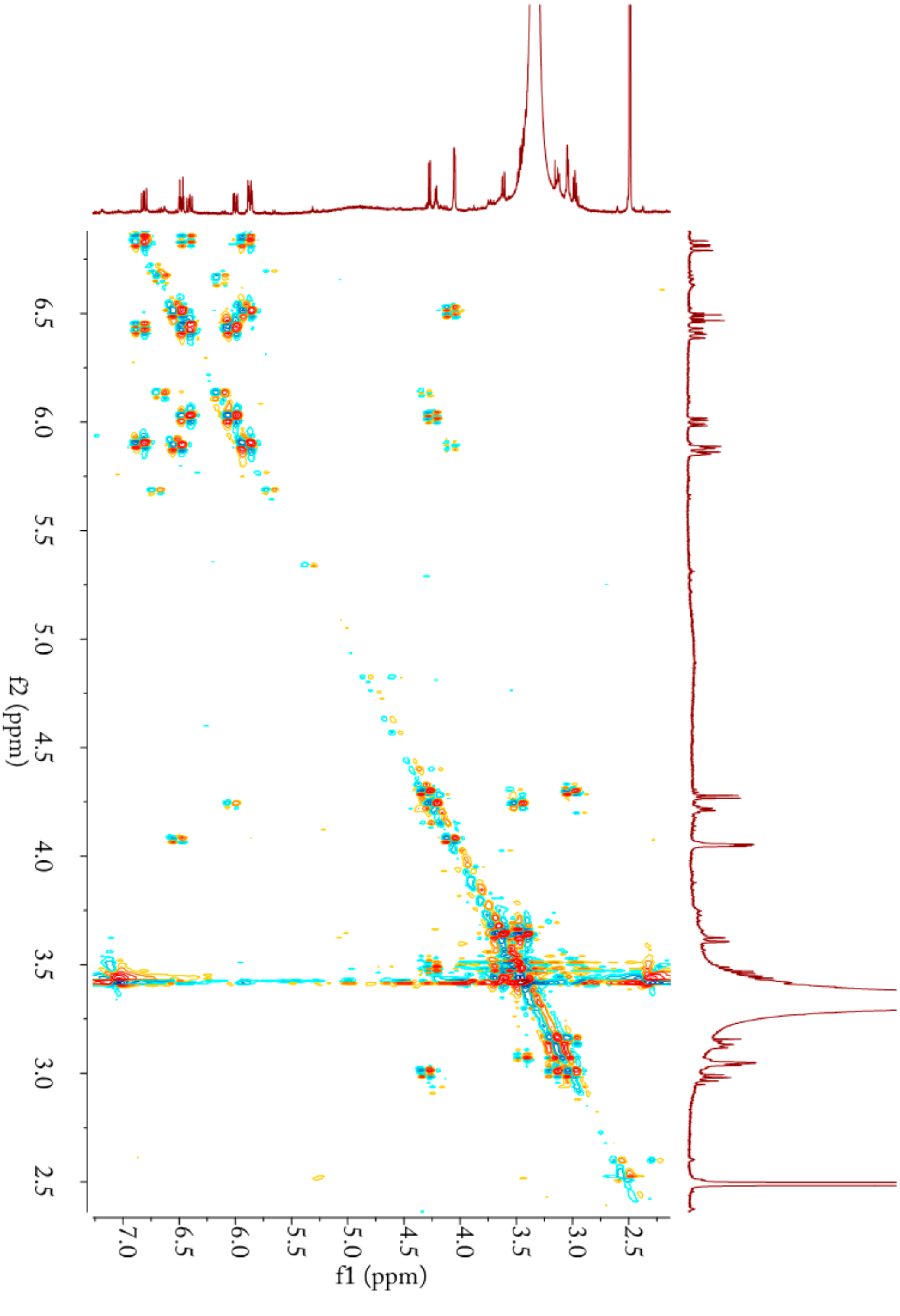


**Figure S7.** $^{1}$H-$^{1}$H COSY spectrum of compound **1** (600 MHz, in DMSO-$d_6$).

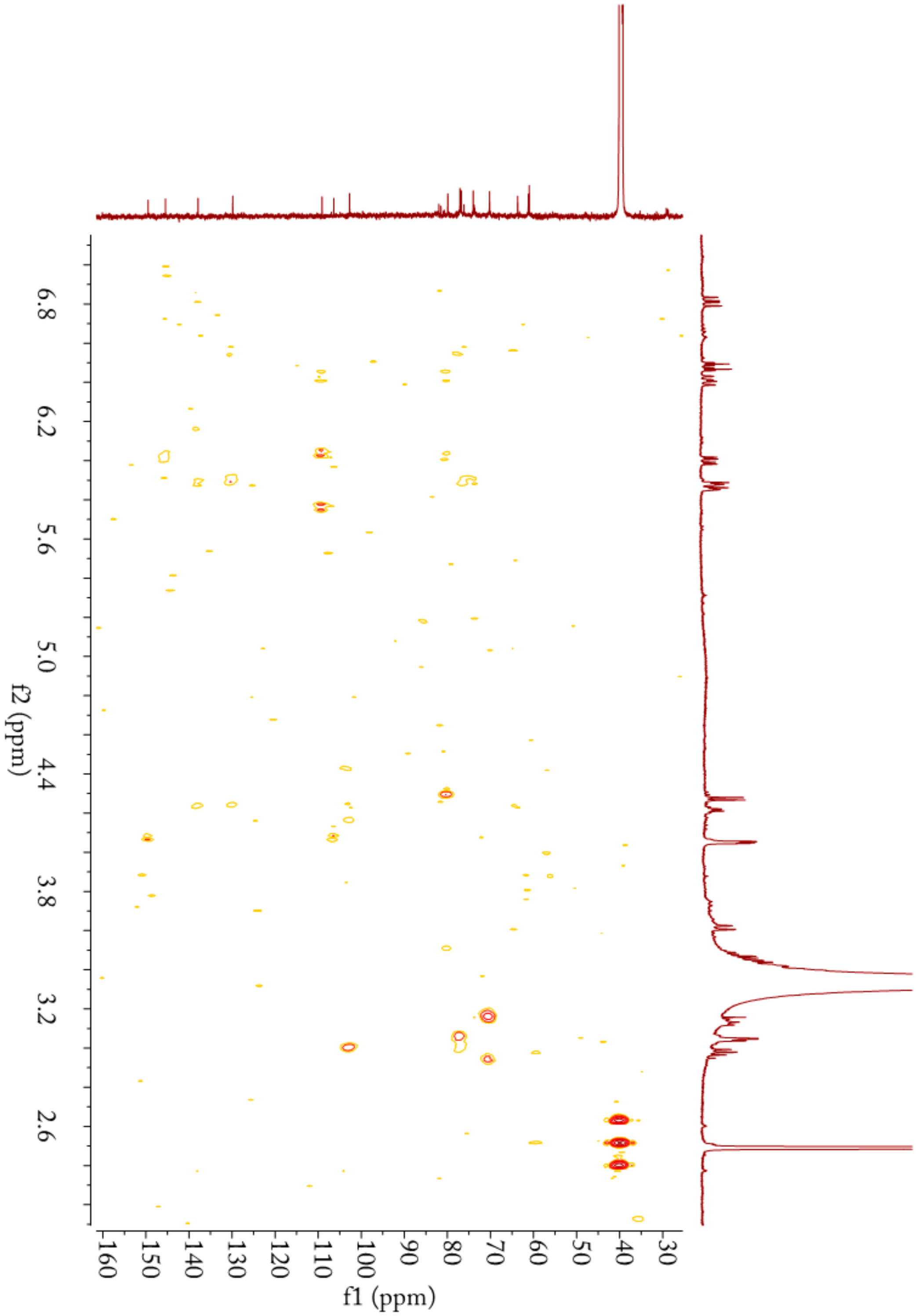


**Figure S8.** HMBC spectrum of compound **1** (600 MHz, in DMSO-$d_6$).

# Qualitative Analysis Report

| | | | |
|---|---|---|---|
| **Data Filename** | BFM-ZAO.d | **Sample Name** | BFM-ZAO |
| **Sample Type** | Sample | **Position** | P1-D7 |
| **Instrument Name** | Instrument 1 | **User Name** | |
| **Acq Method** | TEST-POS-01-WL.m | **Acquired Time** | 11/11/2014 4:26:53 PM |
| **IRM Calibration Status** | Success | **DA Method** | Default.m |
| **Comment** | | | |
| **Data Filename** | BFM-ZAO-NEG.d | **Sample Name** | BFM-ZAO |
| **Sample Type** | Sample | **Position** | P1-D7 |
| **Instrument Name** | Instrument 1 | **User Name** | |
| **Acq Method** | TEST-NEG-01-WL.m | **Acquired Time** | 11/11/2014 4:58:04 PM |
| **IRM Calibration Status** | Success | **DA Method** | Default.m |
| **Comment** | | | |

## User Spectra

| Fragmentor Voltage | Collision Energy | Ionization Mode |
|---|---|---|
| 120 | 0 | Esi |

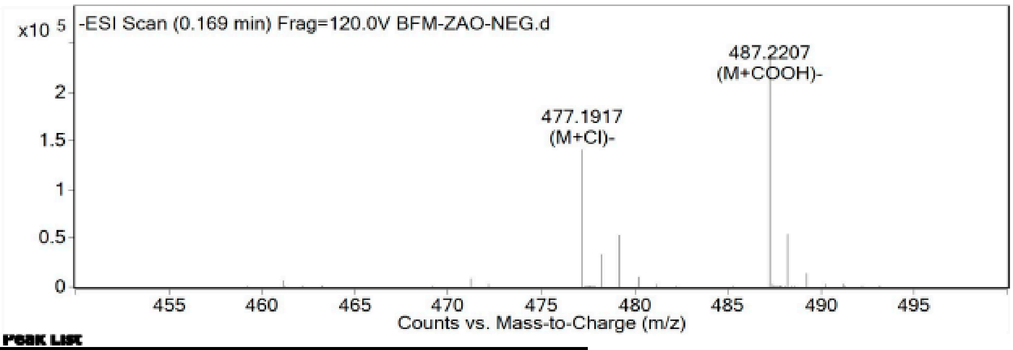


**Peak List**

| m/z | z | Abund | Formula | Ion |
|---|---|---|---|---|
| 68.9962 | | 96757 | | |
| 112.9858 | | 179609 | | |
| 477.1917 | 1 | 142017 | C22 H34 Cl O9 | (M+Cl)- |
| 479.1905 | 1 | 53192 | C22 H34 Cl O9 | (M+Cl)- |
| 487.2207 | 1 | 239659 | C23 H35 O11 | (M+COOH)- |
| 488.2235 | 1 | 53870 | C23 H35 O11 | (M+COOH)- |
| 955.9719 | 1 | 100294 | | |
| 966.0007 | | 50450 | | |
| 982.9899 | | 50093 | | |
| 1033.9868 | | 33735 | | |

**Formula Calculator Element Limits**

| Element | Min | Max |
|---|---|---|
| C | 3 | 60 |
| H | 0 | 120 |
| O | 0 | 20 |

**Formula Calculator Results**

| Formula | Best | Mass | Tgt Mass | Diff (ppm) | Ion Species | Score |
|---|---|---|---|---|---|---|
| C22 H34 O9 | TRUE | 442.2223 | 442.2203 | -4.64 | C22 H34 Cl O9 | 90.72 |
| C22 H34 O9 | TRUE | 442.2225 | 442.2203 | -5.02 | C23 H35 O11 | 88 |

--- End Of Report ---



**Figure S9.** HRESI mass spectrum of compound **2**.

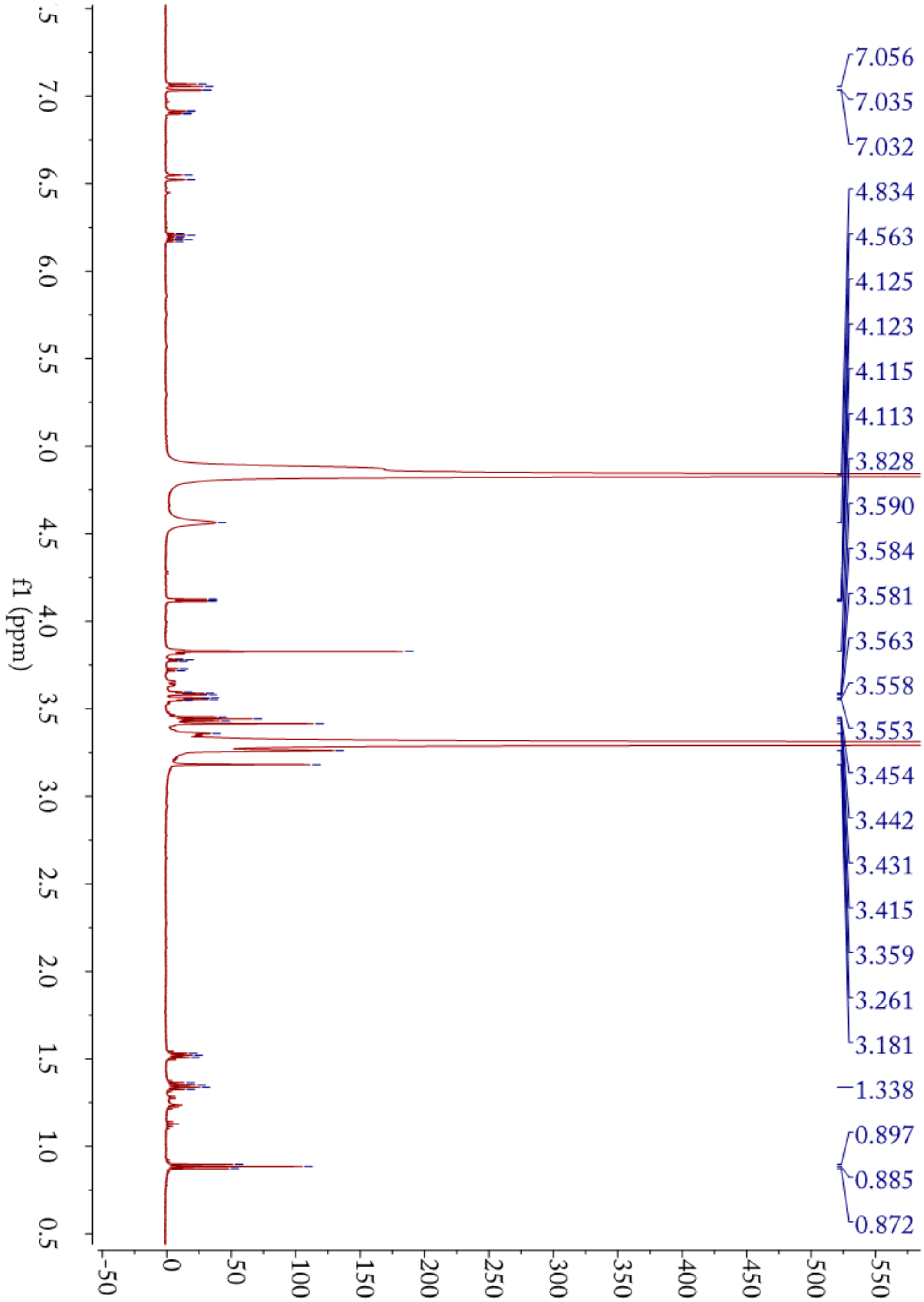


**Figure S10.** $^{1}$H-NMR spectrum of compound **2** (600 MHz, in $CD_3OD$).

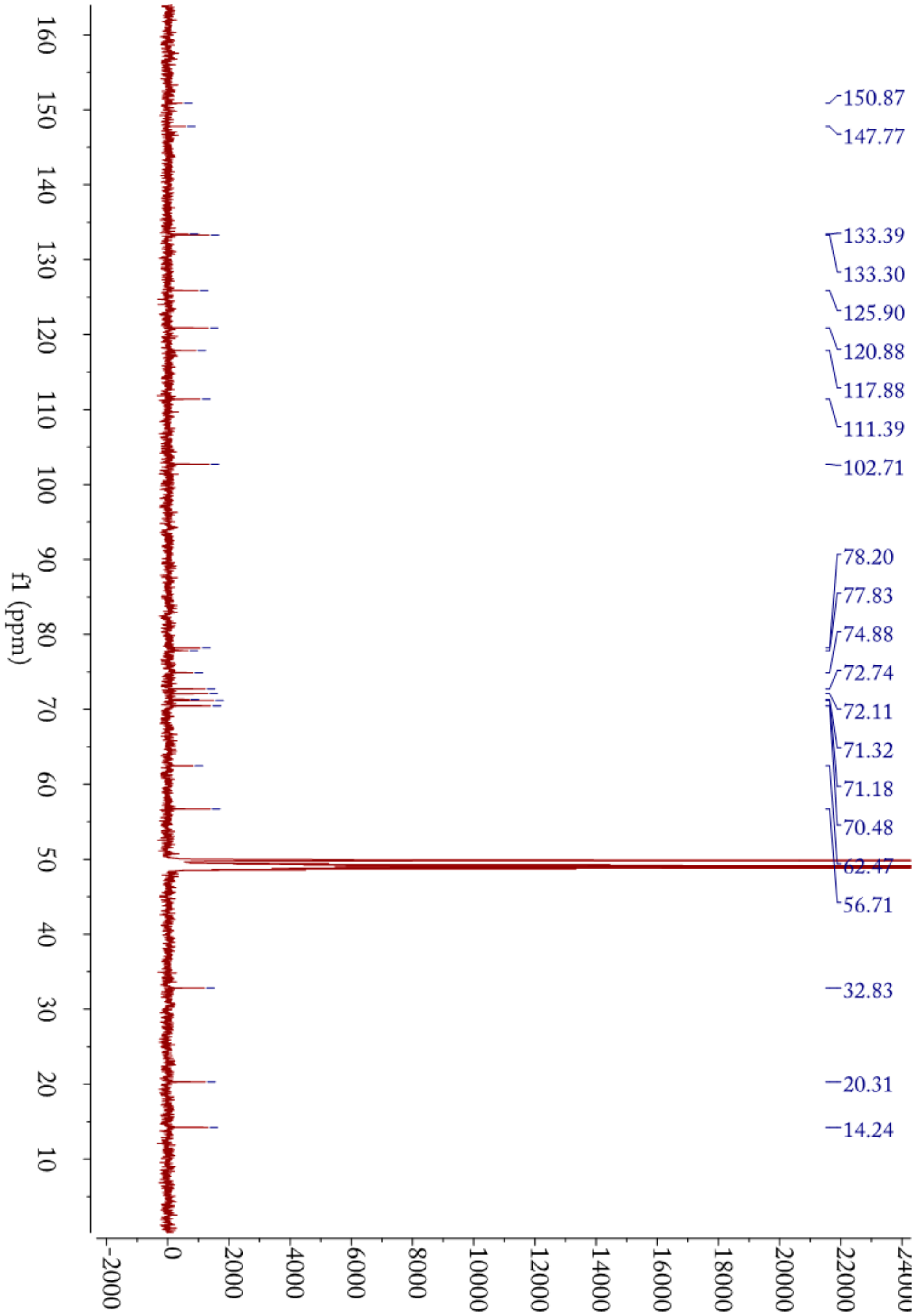


**Figure S11.** $^{13}$C-NMR spectrum of compound **2** (150 MHz, in $CD_3OD$).

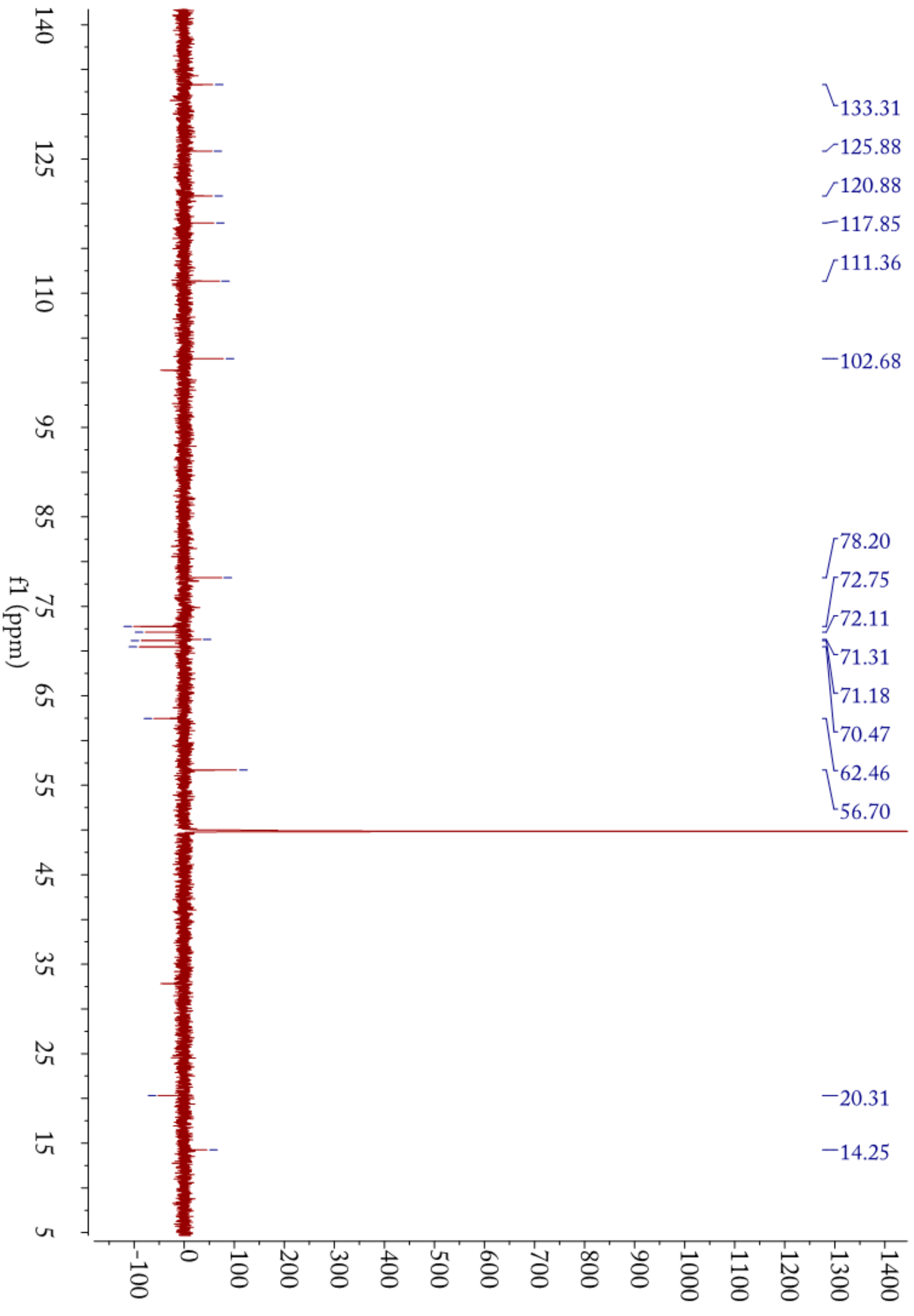


**Figure S12.** DEPT spectrum of compound **2** (150 MHz, in $CD_3OD$).

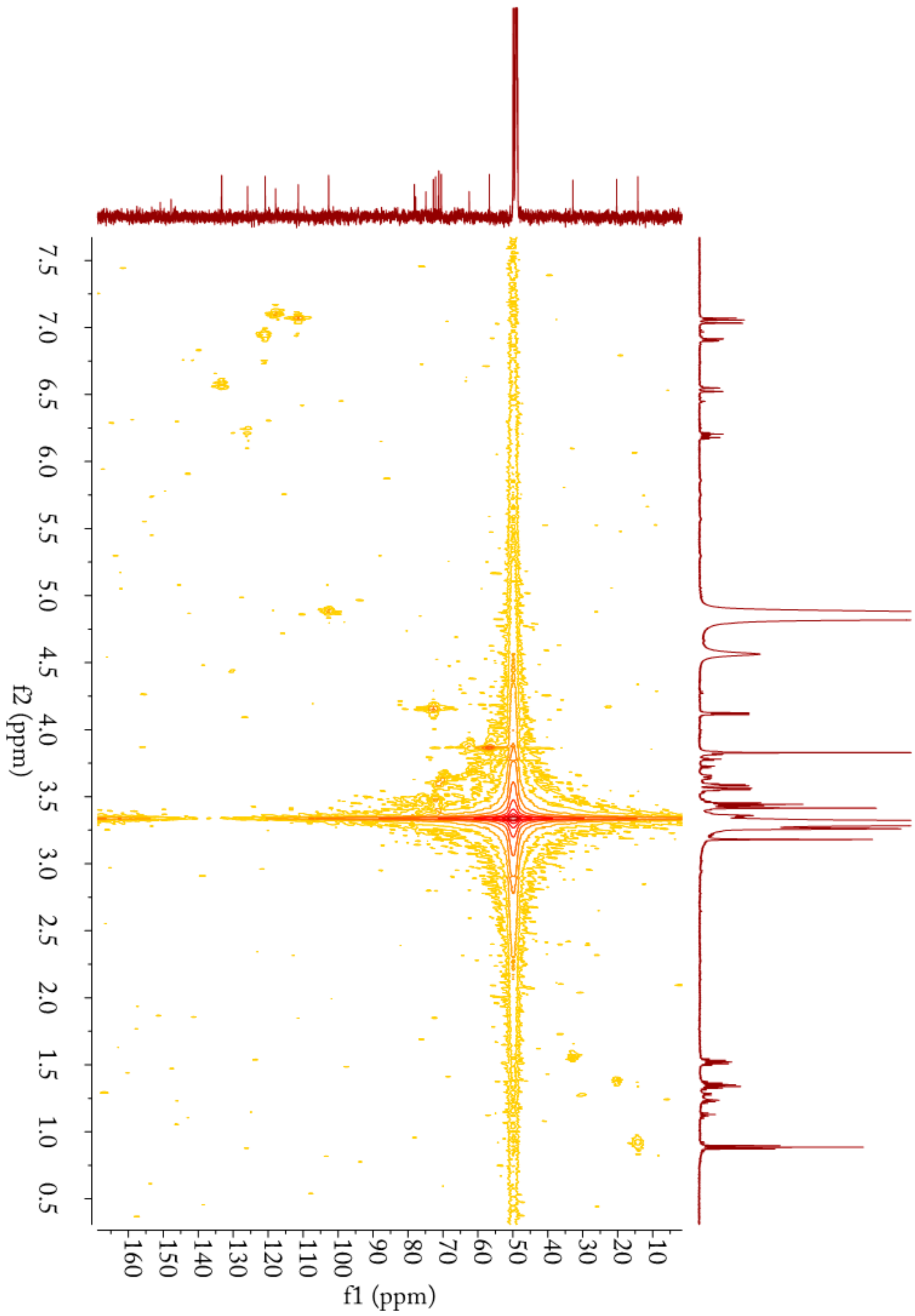


**Figure S13.** HSQC spectrum of compound **2** (600 MHz, in $CD_3OD$).

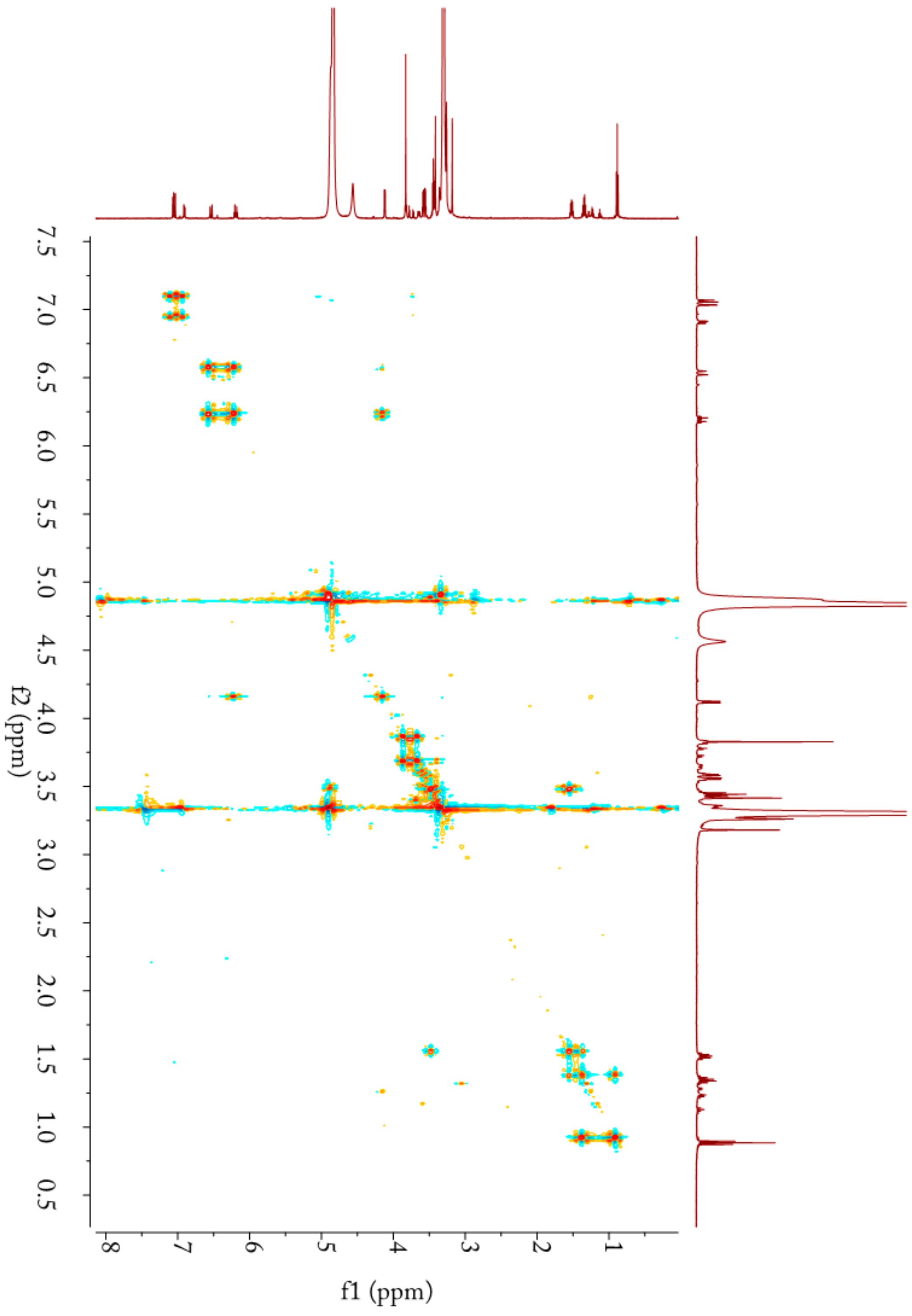


**Figure S14.** $^{1}H$-$^{1}H$ COSY spectrum of compound **2** (600 MHz, in $CD_3OD$).

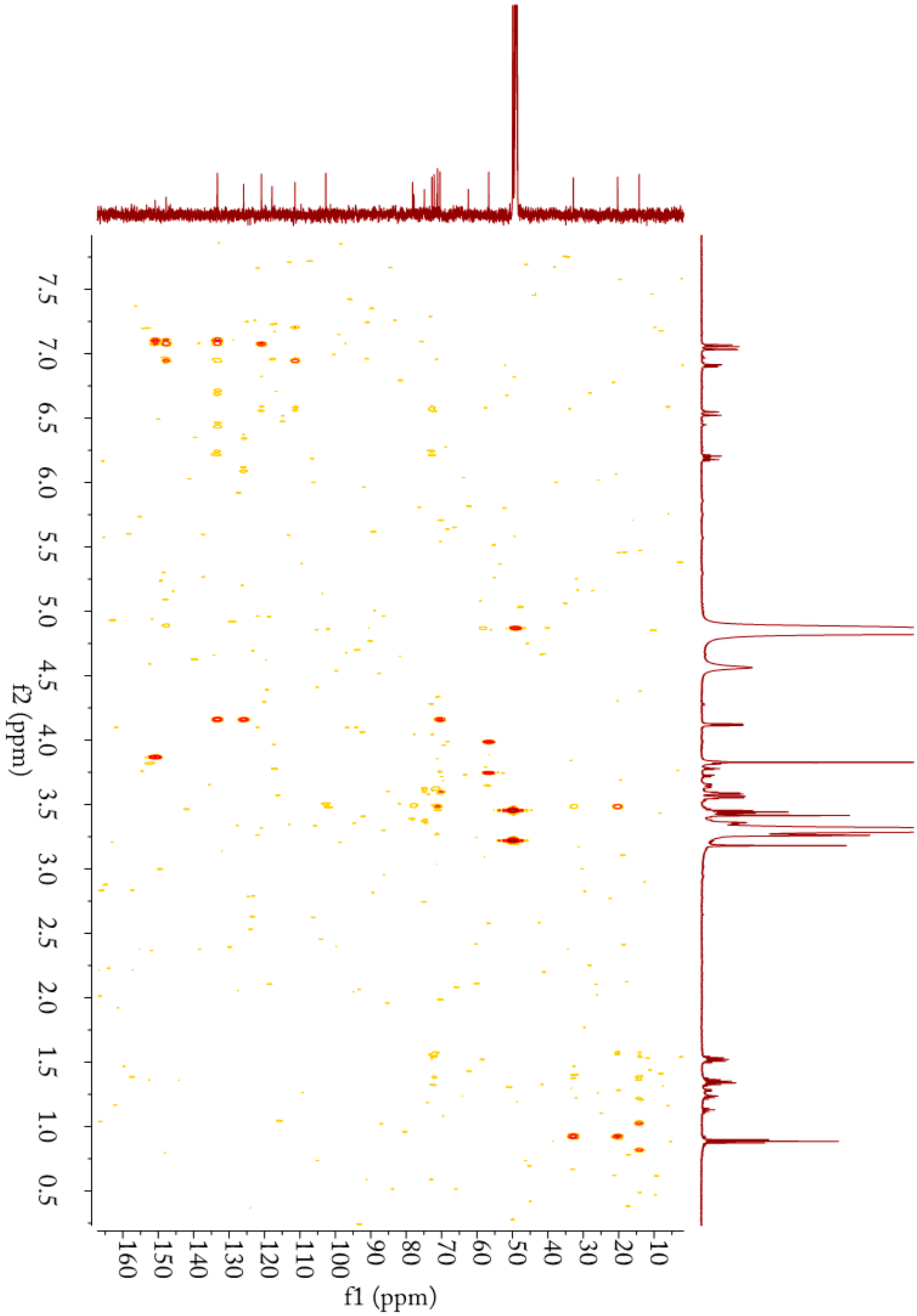


**Figure S15.** HMBC spectrum of compound **2** (600 MHz, in $CD_3OD$).

# Qualitative Analysis Report

| | | | |
|---|---|---|---|
| **Data Filename** | BFA-6.d | **Sample Name** | BFA-6 |
| **Sample Type** | Sample | **Position** | P1-D8 |
| **Instrument Name** | Instrument 1 | **User Name** | |
| **Acq Method** | TEST-POS-01-WL.m | **Acquired Time** | 11/11/2014 4:28:31 PM |
| **IRM Calibration Status** | Success | **DA Method** | Default.m |
| **Comment** | | | |
| **Data Filename** | BFA-6-NEG.d | **Sample Name** | BFA-6 |
| **Sample Type** | Sample | **Position** | P1-D8 |
| **Instrument Name** | Instrument 1 | **User Name** | |
| **Acq Method** | TEST-NEG-01-WL.m | **Acquired Time** | 11/11/2014 4:59:40 PM |
| **IRM Calibration Status** | Success | **DA Method** | Default.m |
| **Comment** | | | |

## User Spectra

| Fragmentor Voltage | Collision Energy | Ionization Mode |
|---|---|---|
| 120 | 0 | Esi |

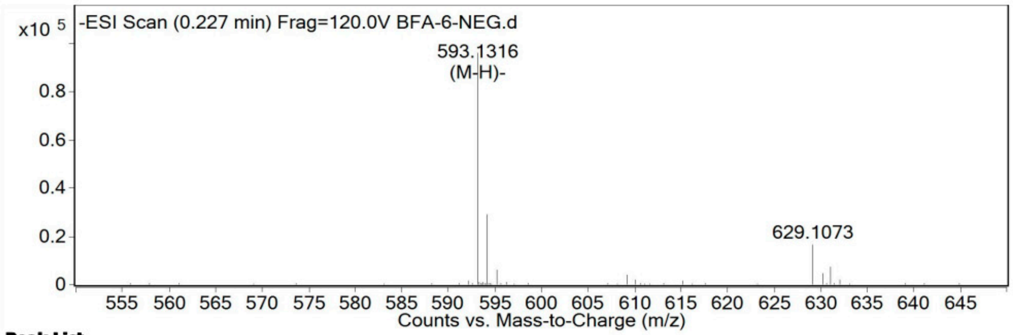


**Peak List**

| *m/z* | z | Abund | Formula | Ion |
|---|---|---|---|---|
| 68.9963 | | 76658 | | |
| 112.9857 | | 146937 | | |
| 160.842 | | 22421 | | |
| 197.8083 | | 20953 | | |
| 593.1316 | 1 | 96289 | C30 H25 O13 | (M-H)- |
| 594.1345 | 1 | 28955 | C30 H25 O13 | (M-H)- |
| 955.9712 | 1 | 56329 | | |
| 966.0007 | 1 | 60958 | | |
| 982.9909 | 1 | 71446 | | |
| 1033.9866 | | 25585 | | |

**Formula Calculator Element Limits**

| Element | Min | Max |
|---|---|---|
| C | 3 | 60 |
| H | 0 | 120 |
| O | 0 | 20 |

**Formula Calculator Results**

| Formula | Best | Mass | Tgt Mass | Diff (ppm) | Ion Species | Score |
|---|---|---|---|---|---|---|
| C30 H26 O13 | TRUE | 594.1389 | 594.1373 | -2.64 | C30 H25 O13 | 94.26 |

| Fragmentor Voltage | Collision Energy | Ionization Mode |
|---|---|---|
| 120 | 0 | Esi |



**Figure S16.** HRESI mass spectrum of compound **3**.

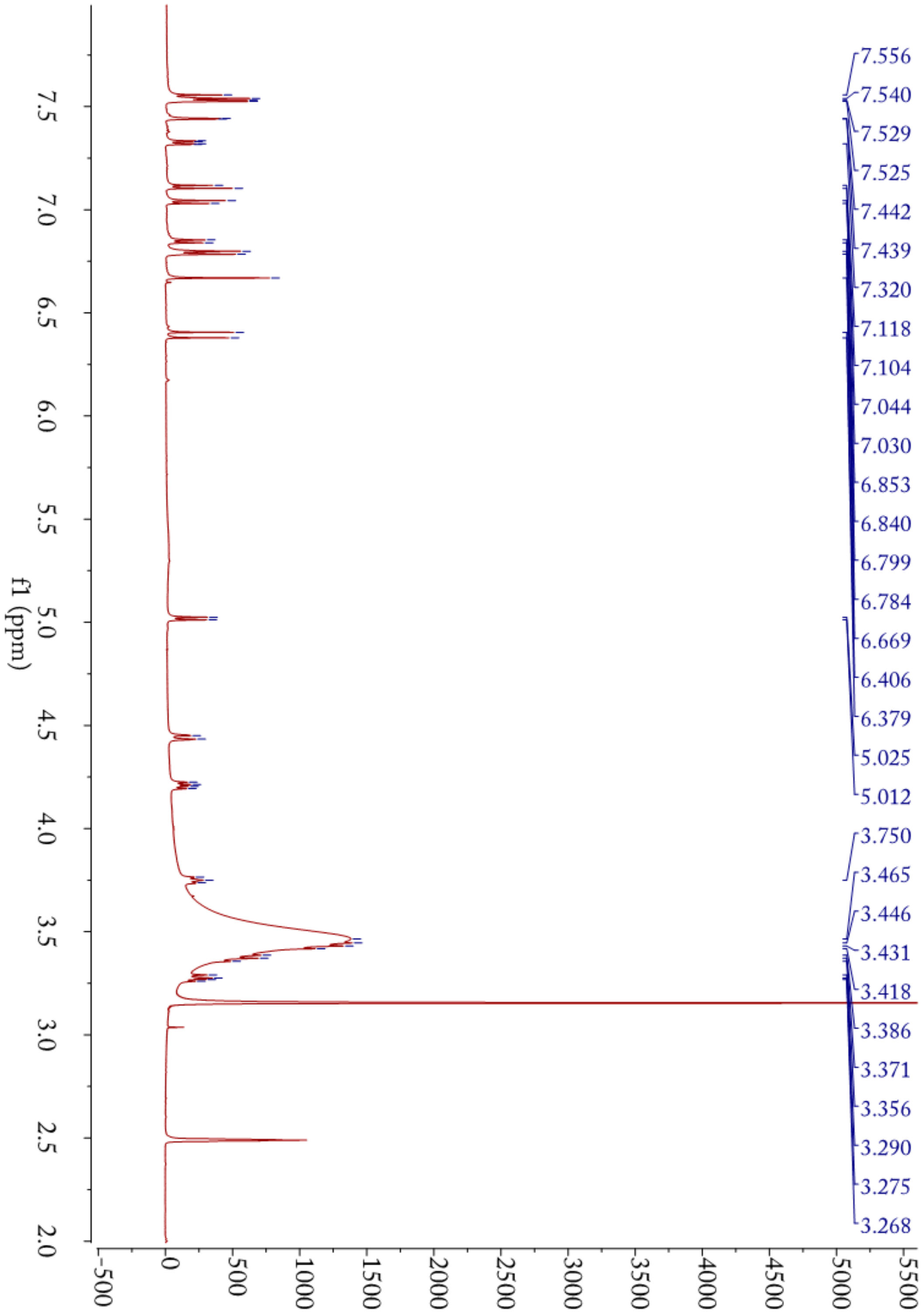

**Figure S17.** $^{1}$H-NMR spectrum of compound **3** (600 MHz, in DMSO-$d_6$).

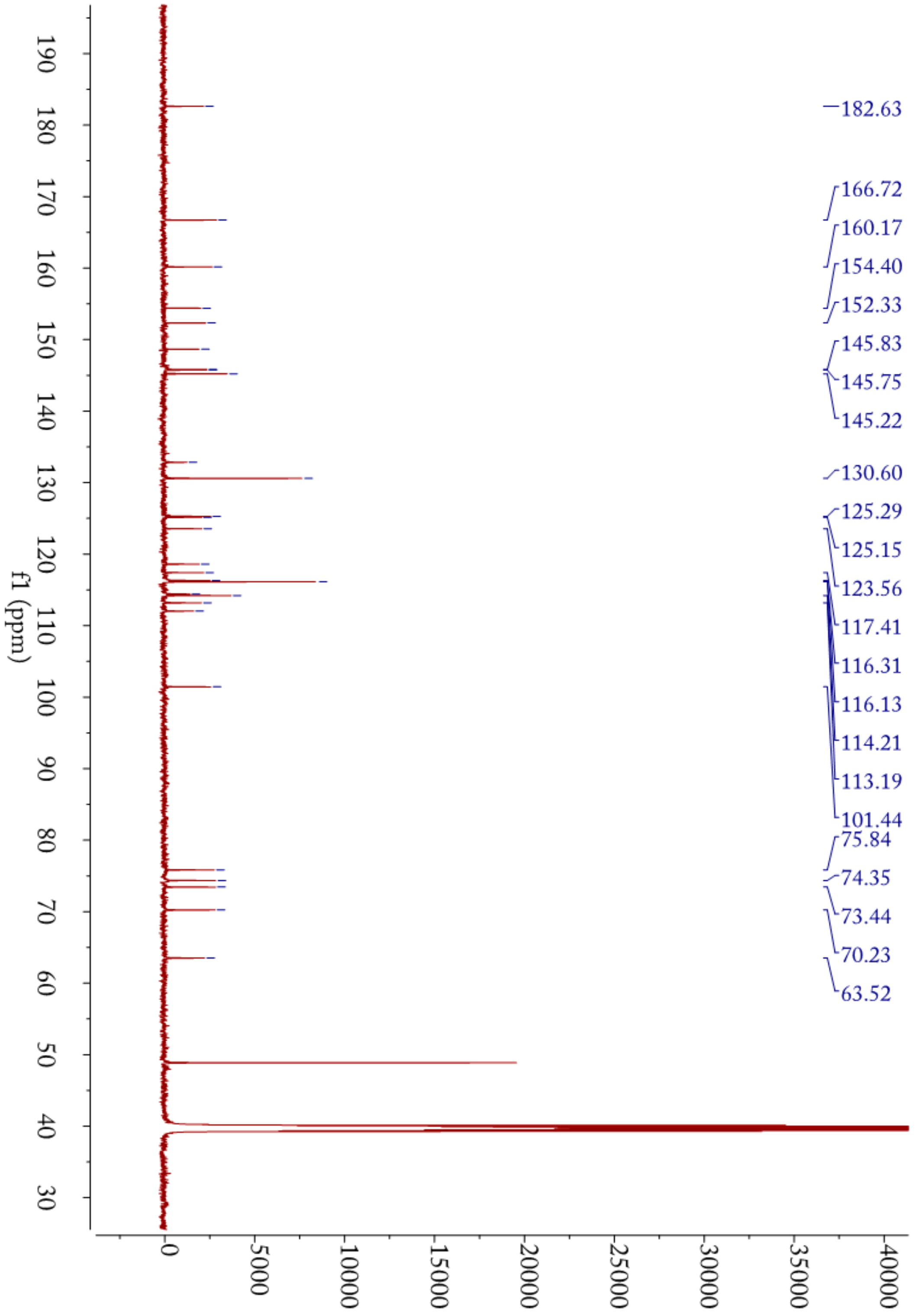


**Figure S18.** $^{13}$C-NMR spectrum of compound **3** (150 MHz, in DMSO-$d_6$).

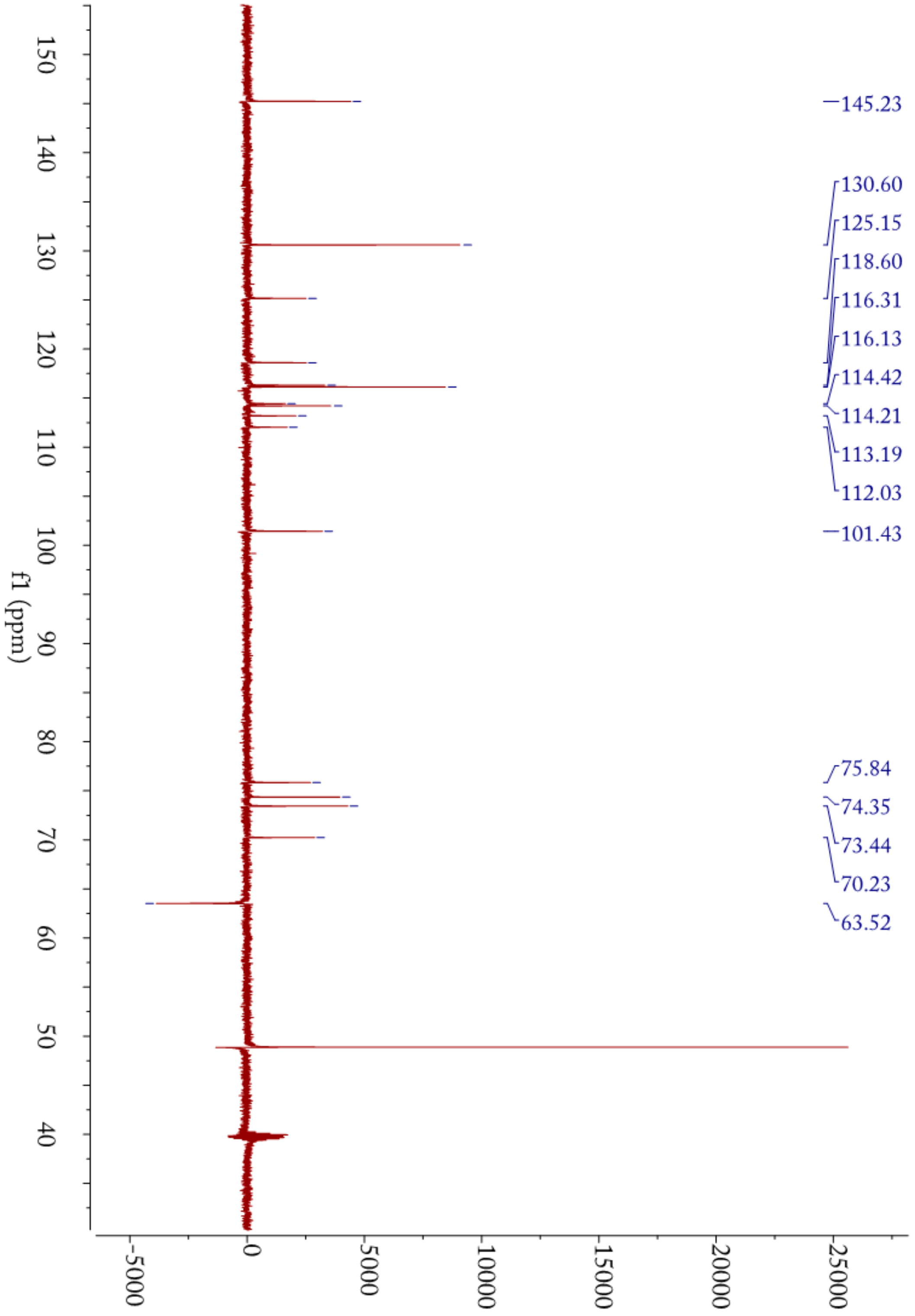


**Figure S19.** DEPT spectrum of compound **3** (150 MHz, in DMSO-$d_6$).

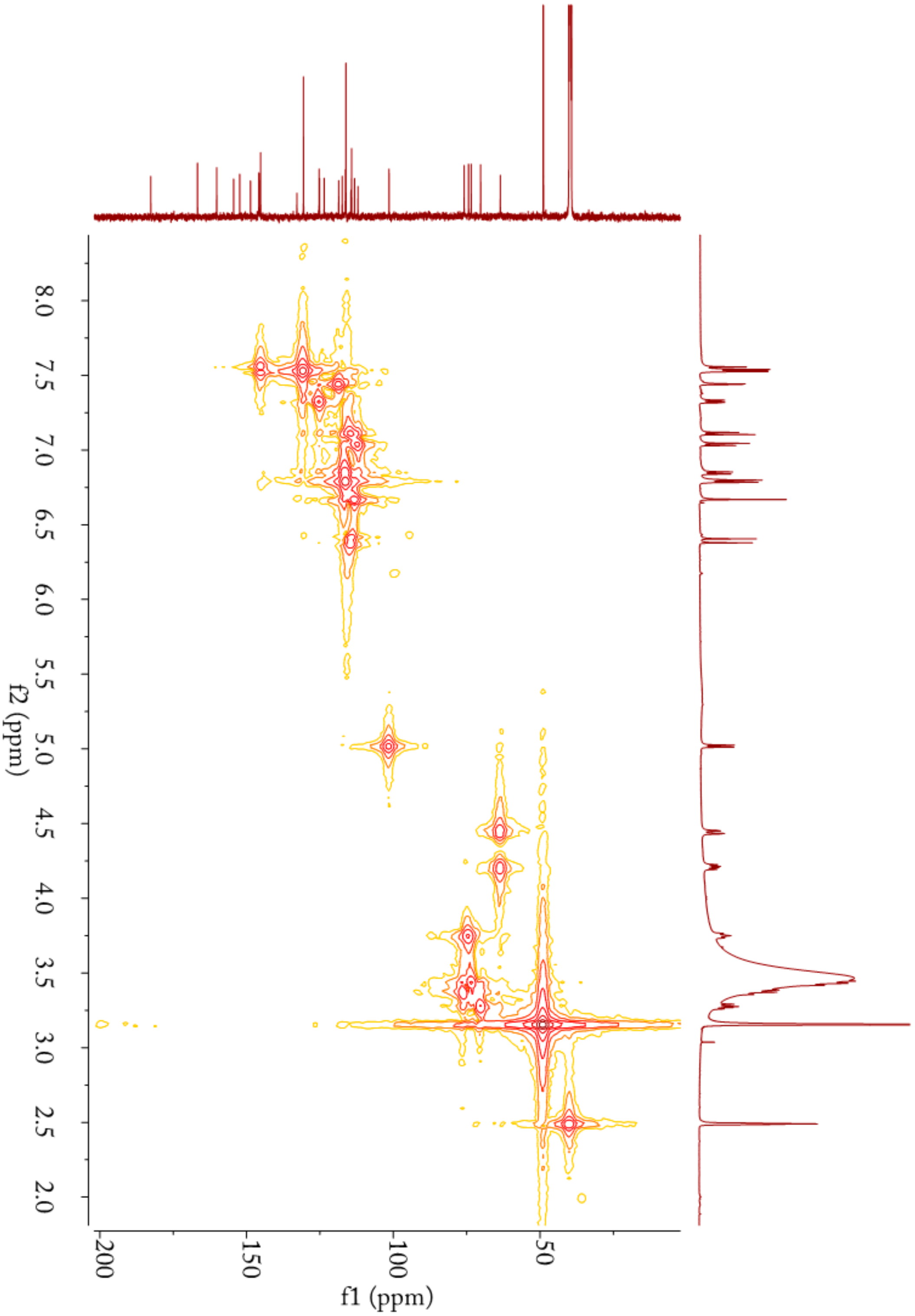


**Figure S20.** HSQC spectrum of compound **3** (600 MHz, in DMSO-$d_6$).

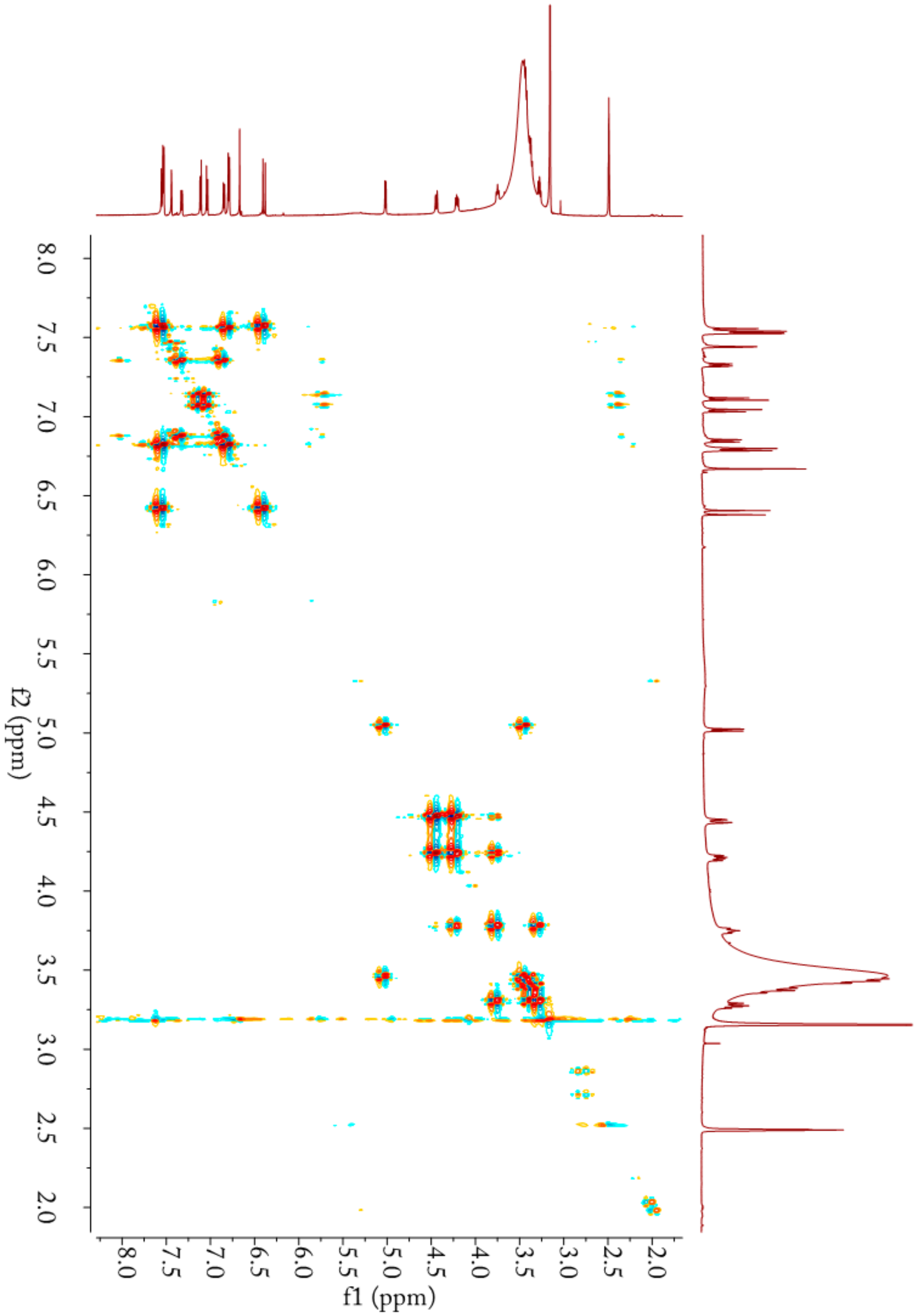


**Figure S21.** $^{1}H$-$^{1}H$ COSY spectrum of compound **3** (600 MHz, in DMSO-*d*$_6$).

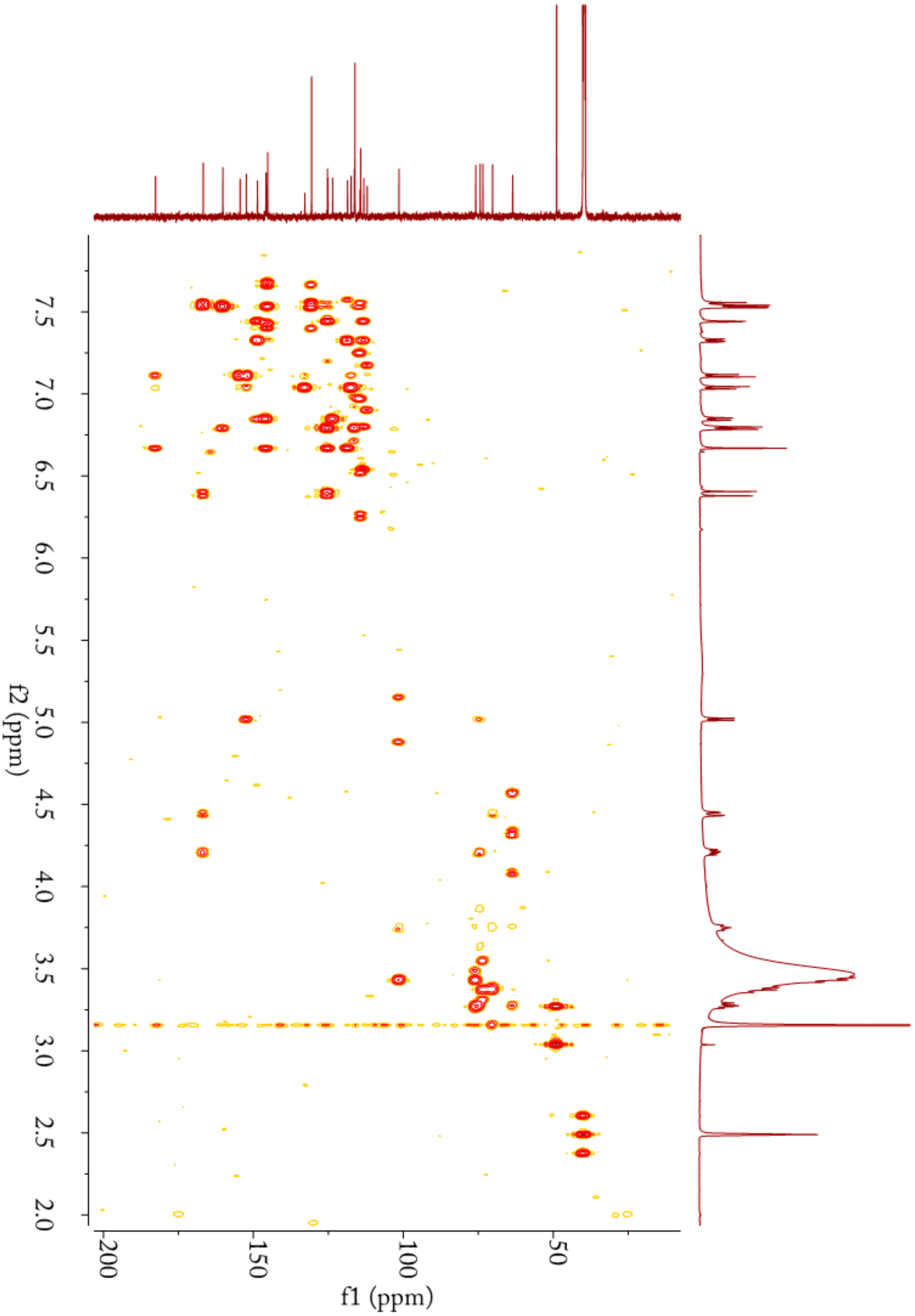


**Figure S22.** HMBC spectrum of compound **3** (600 MHz, in DMSO-$d_6$).

Mobile phase of HPLC-DAD analysis for compounds **1**, **2**, **3**, **9**, **15**, **21**, **24**, **51** and EtOH extract of *B. frondosa* with Diamonsil C18 column (250 mm × 4.6 mm, 5.0 μm, Dikma Technologies, Beijing, China)

| No. | Time (min) | $CH_3OH$ (%) | 0.1% Aqueous Formic Acid (%) | Flow (mL/min) |
|---|---|---|---|---|
| 1 | 0.00 | 10.0 | 90.0 | 0.10 |
| | 30.00 | 100.0 | 0.0 | 0.10 |
| 2 | 0.00 | 20.0 | 80.0 | 0.10 |
| | 80.00 | 100.0 | 0.0 | 0.10 |

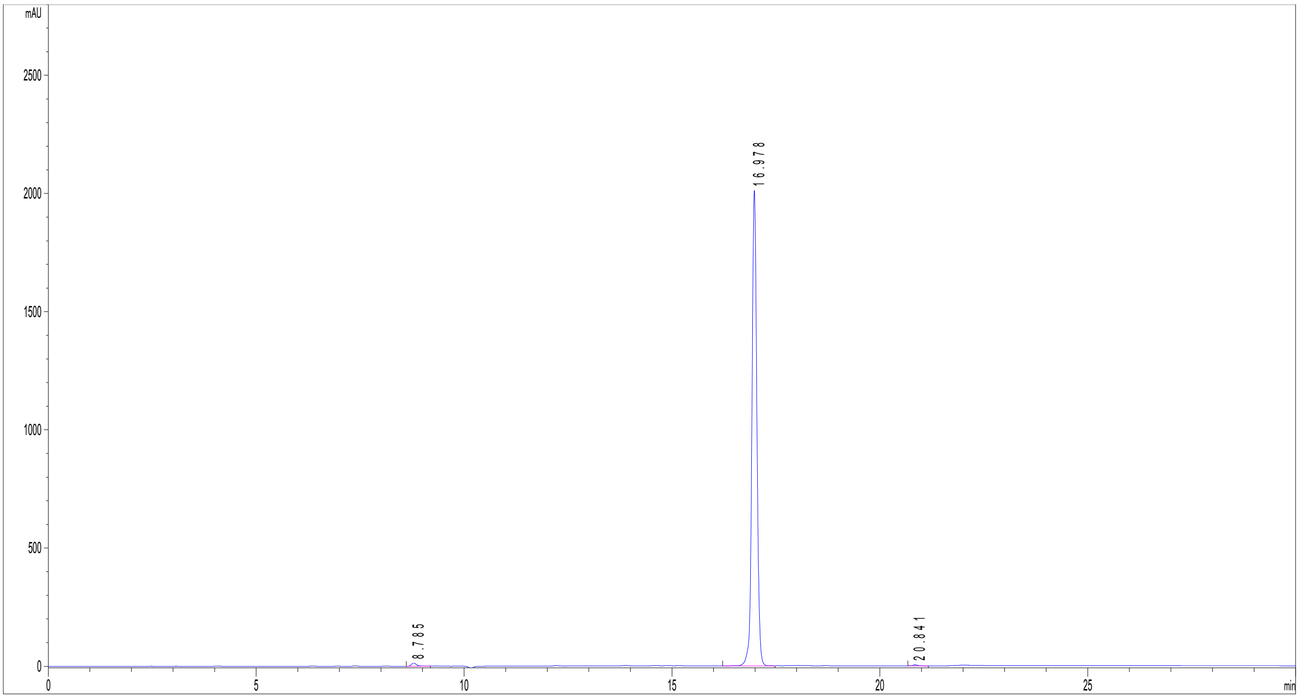


| No. | Time | Area | Height | Width | Area% |
|---|---|---|---|---|---|
| 2 | 16.978 | 16638.5 | 2011 | 0.1265 | 99.057 |

**Figure S23.** HPLC profile of compound 1. Mobile phase of 0 min 10% MeOH–30 min 100% MeOH, Time = 30 min.

**(a)**

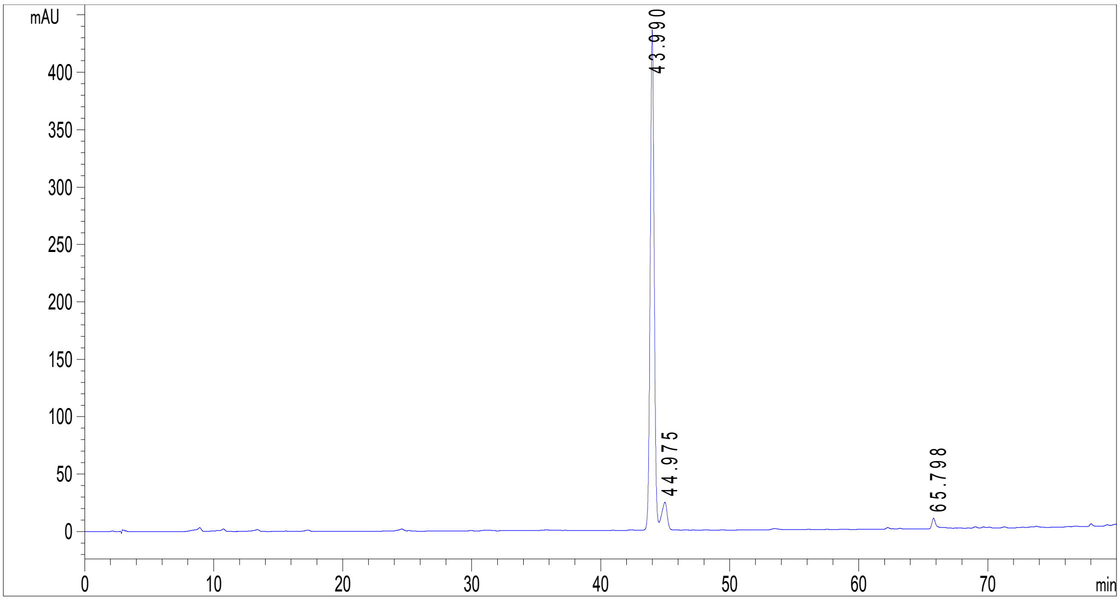


| No. | Time | Area | Height | Width | Area% |
|---|---|---|---|---|---|
| 1 | 43.99 | 9331.9 | 435.9 | 0.3313 | 91.239 |

**(b)**

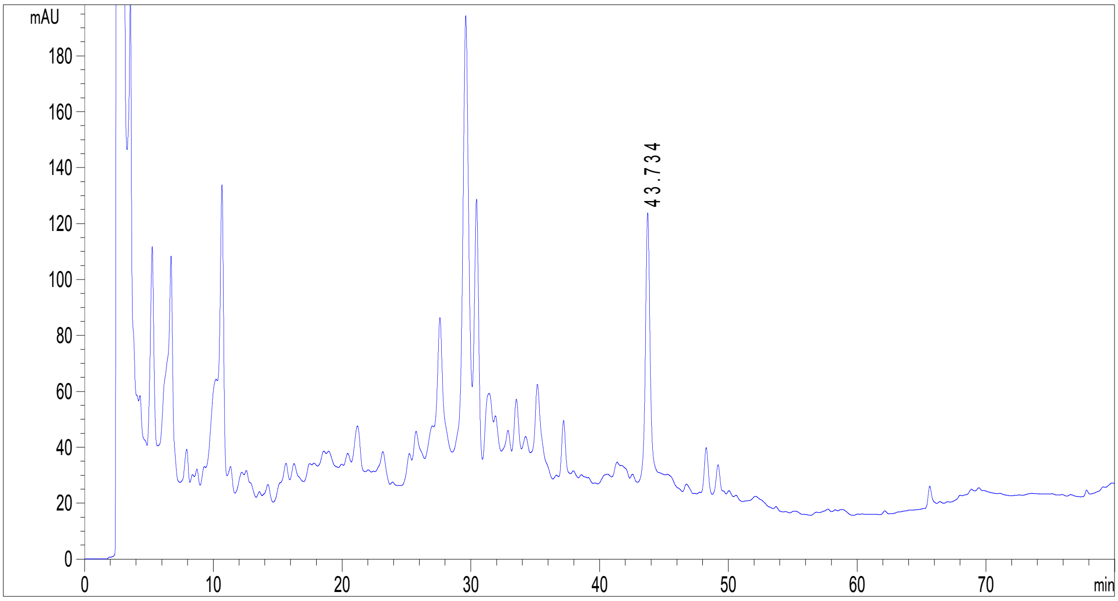


| No. | Time | Area | Height | Width | Area% |
|---|---|---|---|---|---|
| 44 | 43.734 | 2421.2 | 95.3 | 0.3866 | 5.307 |

**Figure S24.** HPLC profiles of compound 2 (**a**) and EtOH extract of *B. frondosa* (**b**). Mobile phase of 0 min 20% MeOH–80 min 100% MeOH, Time = 80 min. Compound **2** is a natural chemical constituent, which is identified by HPLC.

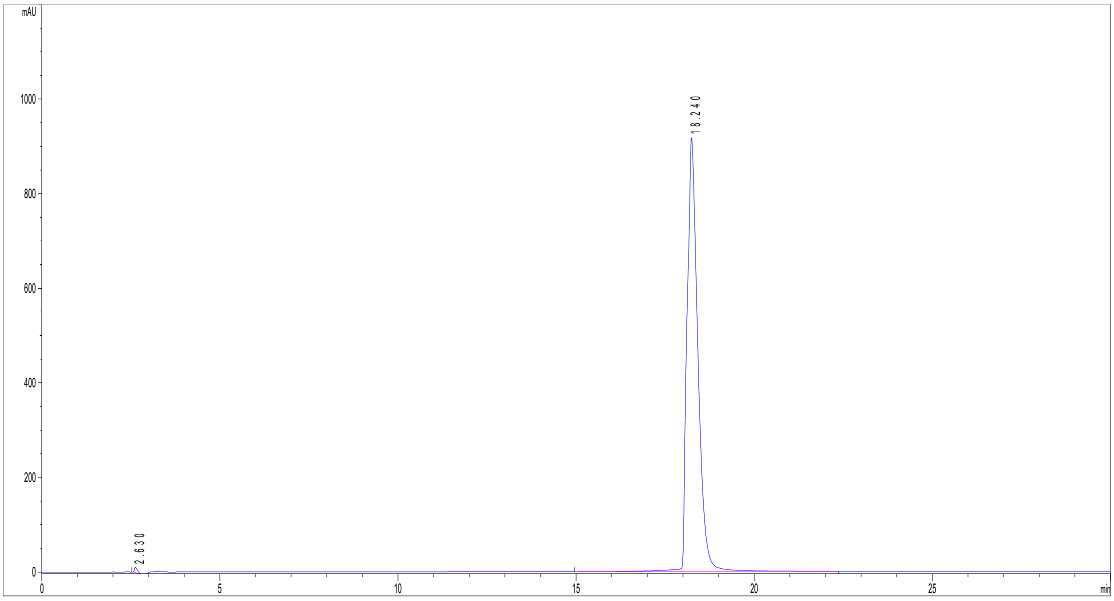


| No. | Time | Area | Height | Width | Area% |
|---|---|---|---|---|---|
| 2 | 18.24 | 19459 | 917.3 | 0.2955 | 99.662 |

**Figure S25.** HPLC profile of compound **3**. Mobile phase of 0 min 10% MeOH–30 min 100% MeOH, Time = 30 min.

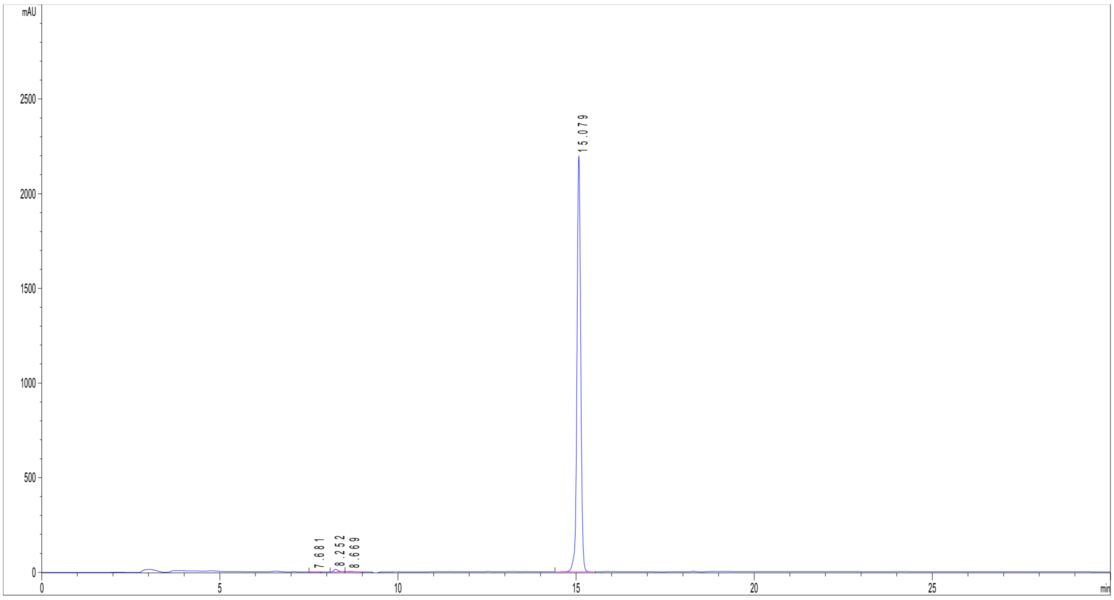


| No. | Time | Area | Height | Width | Area% |
|---|---|---|---|---|---|
| 4 | 15.079 | 16115.4 | 2195.6 | 0.1136 | 99.167 |

**Figure S26.** HPLC profile of compound 9. Mobile phase of 0 min 10% MeOH–30 min 100% MeOH, Time = 30 min.

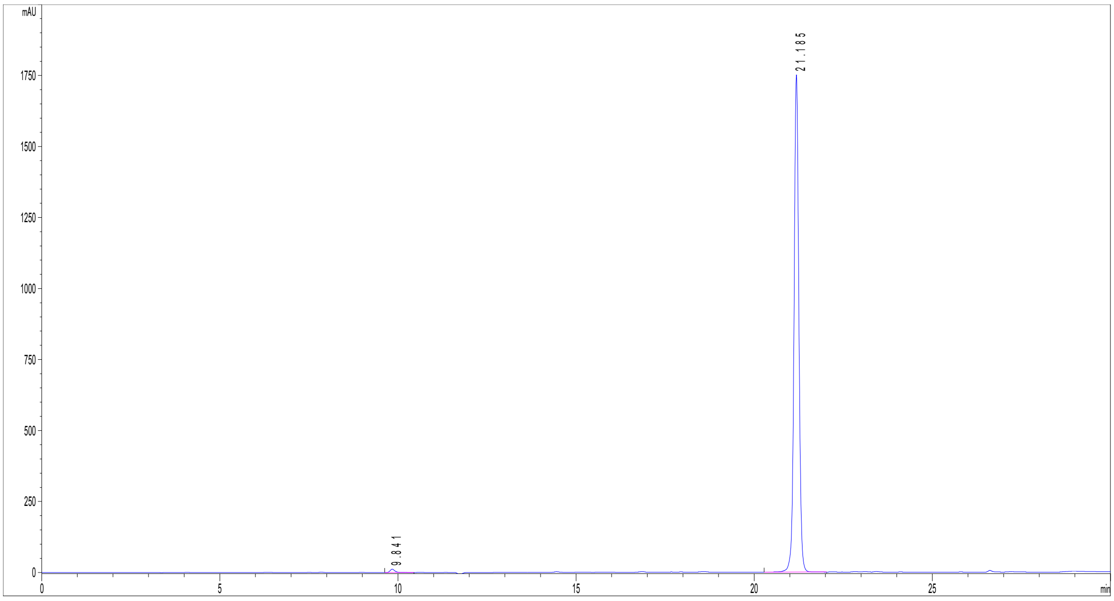


| No. | Time | Area | Height | Width | Area% |
|---|---|---|---|---|---|
| 2 | 21.185 | 16489.1 | 1751 | 0.1459 | 99.293 |

**Figure S27.** HPLC profile of compound **15**. Mobile phase of 0 min 10% MeOH–30 min 100% MeOH, Time =30 min.

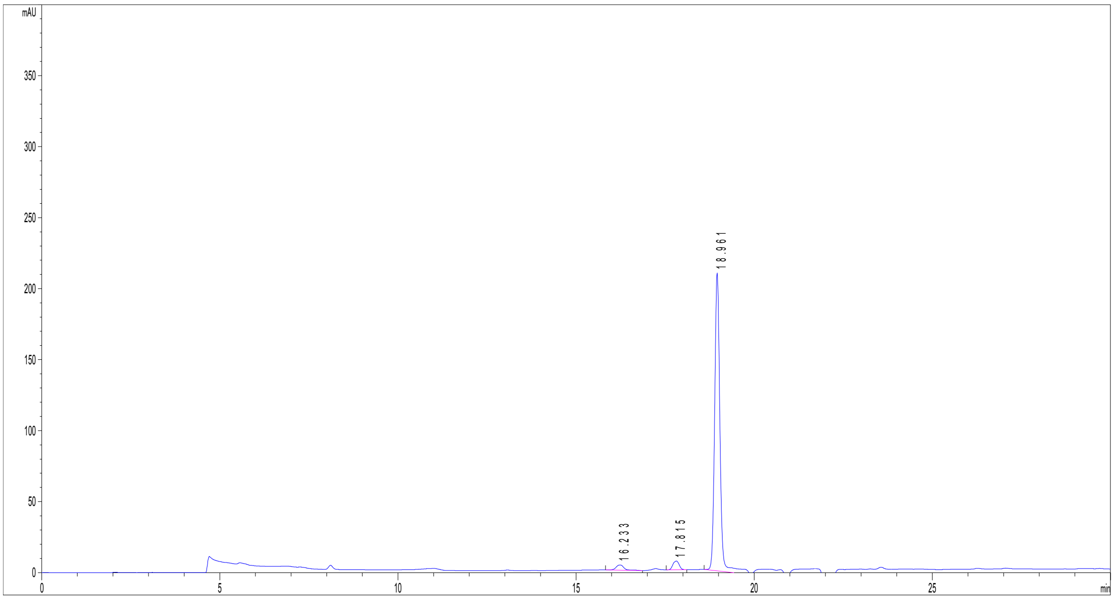


| No. | Time | Area | Height | Width | Area% |
|---|---|---|---|---|---|
| 3 | 18.961 | 2189.7 | 209.8 | 0.1597 | 94.127 |

**Figure S28.** HPLC profile of compound **21**. Mobile phase of 0 min 10% MeOH–30 min 100% MeOH, Time =30 min.

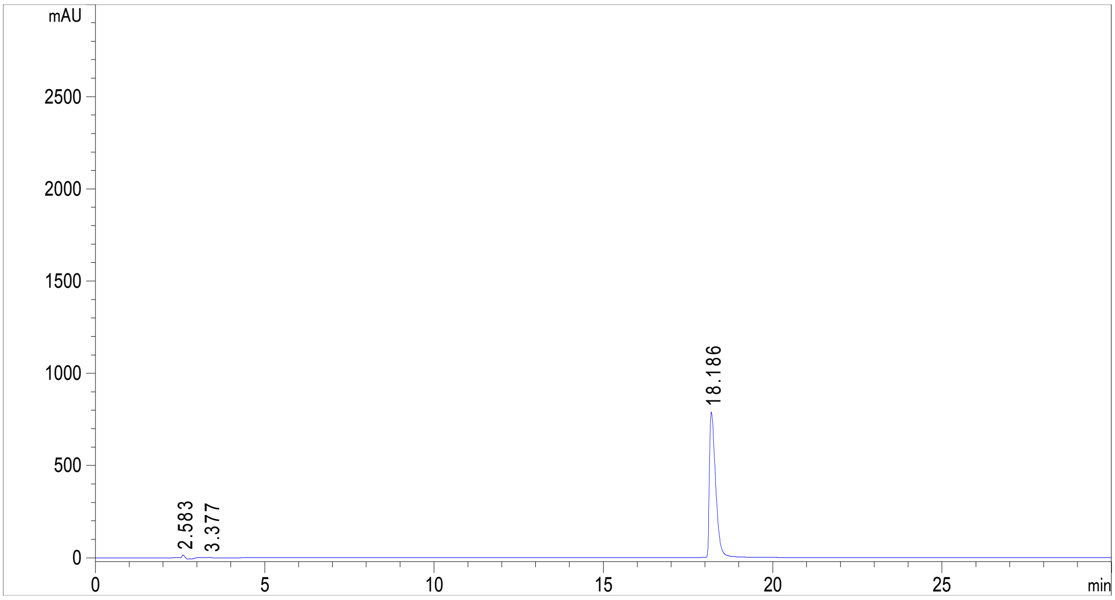


| No. | Time | Area | Height | Width | Area% |
|---|---|---|---|---|---|
| 3 | 18.186 | 10208.7 | 790.4 | 0.1967 | 97.939 |

**Figure S29.** HPLC profile of compound **24**. Mobile phase of 0 min 10% MeOH–30 min 100% MeOH, Time =30 min.

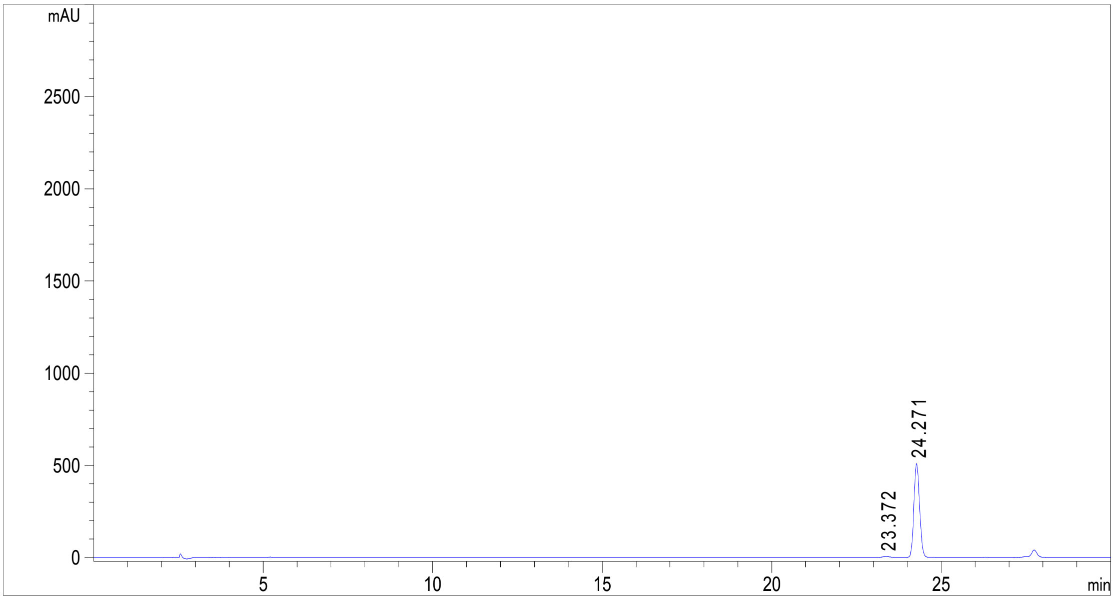


| No. | Time | Area | Height | Width | Area% |
|---|---|---|---|---|---|
| 2 | 24.271 | 6150.1 | 565.5 | 0.179 | 97.089 |

**Figure S30.** HPLC profile of compound **51**. Mobile phase of 0 min 10% MeOH–30 min 100% MeOH, Time =30 min.

**Table S1.** Inhibitory effects of compounds **1–34**, **36**, **39**, **43**, **47**, **51**, **52** (1, 10, 100 μg/mL) on NF-κB in luciferase activity assay.

| Compound | Cell | LPS | Compound + LPS | | |
|---|---|---|---|---|---|
| | | | C-1 μg/mL | C-10 μg/mL | C-100 μg/mL |
| **1** | 2132.00 ± 281.13 ** | 73268.00 ± 4999.01 | 65479.00 ± 2675.95 | 62319.67 ± 4230.77 * | 59421.67 ± 4993.19 * |
| **2** | 2132.00 ± 281.13 ** | 73268.00 ± 4999.01 | 66488.00 ± 3148.37 | 62489.67 ± 3724.55 * | 56398.67 ± 4119.77 |
| **3** | 2132.00 ± 281.13 ** | 73268.00 ± 4999.01 | 68896.00 ± 3696.88 | 62387.67 ± 3843.46 * | 59076.33 ± 6732.61 * |
| **4** | 2132.00 ± 281.13 ** | 73268.00 ± 4999.01 | 68333.00 ± 5552.84 | 65038.67 ± 5021.67 | 62632.00 ± 4289.43 * |
| **5** | 2132.00 ± 281.13 ** | 73268.00 ± 4999.01 | 69027.00 ± 4565.20 | 61769.67 ± 4480.88 * | 58630.67 ± 2551.23 ** |
| **6** | 2132.00 ± 281.13 ** | 73268.00 ± 4999.01 | 68977.33 ± 4635.98 | 65313.00 ± 3016.18 | 60990.67 ± 3209.34 * |
| **7** | 2132.00 ± 281.13 ** | 73268.00 ± 4999.01 | 68038.67 ± 4100.52 | 67432.00 ± 6221.41 | 64396.67 ± 5314.31 |
| **8** | 2132.00 ± 281.13 ** | 73268.00 ± 4999.01 | 69060.00 ± 4137.77 | 64708.00 ± 2633.98 | 60614.67 ± 4016.71 * |
| **9** | 2132.00 ± 281.13 ** | 73268.00 ± 4999.01 | 62253.33 ± 3875.6 * | 56677.33 ± 5258.80 * | 45549.67 ± 8023.69 ** |
| **10** | 2132.00 ± 281.13 ** | 73268.00 ± 4999.01 | 67950.33 ± 4247.37 | 67385.67 ± 3348.19 | 58837.00 ± 6131.49 * |
| **11** | 2132.00 ± 281.13 ** | 73268.00 ± 4999.01 | 65528.00 ± 4876.20 | 64122.33 ± 3531.52 | 59963.67 ± 3575.50 * |
| **12** | 2132.00 ± 281.13 ** | 73268.00 ± 4999.01 | 67828.33 ± 4365.55 | 63674.33 ± 4035.83 | 53432.00 ± 4547.24 ** |
| **13** | 2132.00 ± 281.13 ** | 73268.00 ± 4999.01 | 69319.33 ± 4163.59 | 65280.67 ± 4343.88 | 56975.67 ± 5995.17 * |
| **14** | 2132.00 ± 281.13 ** | 73268.00 ± 4999.01 | 68371.33 ± 5603.29 | 63247.67 ± 3694.65 * | 58347.33 ± 4447.21 * |
| **15** | 2132.00 ± 281.13 ** | 73268.00 ± 4999.01 | 59902.00 ± 5734.5 * | 58715.33 ± 4789.37 * | 35417.00 ± 7285.47 ** |
| **16** | 2132.00 ± 281.13 ** | 73268.00 ± 4999.01 | 69980.67 ± 4109.9 | 67088.67 ± 2854.42 | 63264.67 ± 4006.62 |
| **17** | 2132.00 ± 281.13 ** | 73268.00 ± 4999.01 | 68069.33 ± 2254.6 | 64314.67 ± 3788.37 | 59662.67 ± 4631.43* |
| **18** | 2132.00 ± 281.13 ** | 73268.00 ± 4999.01 | 65444.67 ± 2904.9 | 60307.00 ± 3656.06 * | 56705.00 ± 5944.80* |
| **19** | 2132.00 ± 281.13 ** | 73268.00 ± 4999.01 | 65878.00 ± 3992.27 | 64450.67 ± 3680.85 | 55090.33 ± 4504.16** |
| **20** | 2132.00 ± 281.13 ** | 73268.00 ± 4999.01 | 69457.00 ± 2955.76 | 65315.33 ± 2724.70 | 58030.00 ± 5092.78 * |
| **21** | 2132.00 ± 281.13 ** | 73268.00 ± 4999.01 | 61918.33 ± 3548.9 * | 50924.00 ± 3502.17 ** | 45476.00 ± 8498.07 ** |
| **22** | 2132.00 ± 281.13 ** | 73268.00 ± 4999.01 | 58070.00 ± 4334.8 * | 51643.33 ± 6534.49 * | 40122.67 ± 4886.15 ** |
| **23** | 2132.00 ± 281.13 ** | 73268.00 ± 4999.01 | 65622.00 ± 3349.58 | 66220.33 ± 5212.61 | 59923.33 ± 6084.11 * |
| **24** | 2132.00 ± 281.13 ** | 73268.00 ± 4999.01 | 65046.00 ± 4545.51 | 64802.33 ± 4082.00 | 59672.67 ± 3753.96 * |
| **25** | 2132.00 ± 281.13** | 73268.00 ± 4999.01 | 66629.67 ± 3227.20 | 63780.33 ± 4066.85 | 63819.67 ± 4675.25 |

**Table S1.** *Cont.*

| Compound | Cell | LPS | Compound + LPS | | |
|---|---|---|---|---|---|
| | | | C-1 µg/mL | C-10 µg/mL | C-100 µg/mL |
| **26** | 2132.00 ± 281.13 ** | 73268.00 ± 4999.01 | 65229.67 ± 4410.53 | 64029.00 ± 4013.51 | 59010.00 ± 5472.91 * |
| **27** | 2132.00 ± 281.13 ** | 73268.00 ± 4999.01 | 66429.00 ± 3131.58 | 60575.00 ± 4677.45 * | 57566.33 ± 4433.86 * |
| **28** | 2132.00 ± 281.13 ** | 73268.00 ± 4999.01 | 67093.00 ± 3899.88 | 65215.00 ± 2716.62 | 59226.00 ± 5025.35 * |
| **29** | 2132.00 ± 281.13 ** | 73268.00 ± 4999.01 | 67276.00 ± 3507.74 | 63942.33 ± 3294.88 | 59497.33 ± 4052.66 * |
| **30** | 2132.00 ± 281.13 ** | 73268.00 ± 4999.01 | 67418.00 ± 4181.95 | 64804.67 ± 3725.58 | 59233.67 ± 4645.91 * |
| **31** | 2132.00 ± 281.13 ** | 73268.00 ± 4999.01 | 67722.33 ± 2772.07 | 64602.67 ± 3300.97 | 58969.00 ± 4565.26 * |
| **32** | 2132.00 ± 281.13 ** | 73268.00 ± 4999.01 | 69157.00 ± 4731.34 | 63617.00 ± 4047.05 | 59162.67 ± 4481.74 * |
| **33** | 2132.00 ± 281.13 ** | 73268.00 ± 4999.01 | 67359.00 ± 3514.87 | 64555.67 ± 4021.52 | 56640.00 ± 4919.46 * |
| **34** | 2132.00 ± 281.13 ** | 73268.00 ± 4999.01 | 68007.67 ± 4107.68 | 63242.33 ± 4638.23 | 52565.67 ± 4279.69 ** |
| **36** | 2132.00 ± 281.13 ** | 73268.00 ± 4999.01 | 70055.33 ± 4111.44 | 64733.33 ± 4174.56 | 59247.00 ± 4648.29 * |
| **39** | 2132.00 ± 281.13 ** | 73268.00 ± 4999.01 | 67420.00 ± 3259.34 | 62314.00 ± 4492.91 * | 57646.67 ± 3659.60 * |
| **43** | 2132.00 ± 281.13 ** | 73268.00 ± 4999.01 | 66801.00 ± 3696.52 | 64209.3 ± 3814.68 | 59015.00± 4434.44 * |
| **47** | 2132.00 ± 281.13 ** | 73268.00 ± 4999.01 | 68683.33 ± 4069.28 | 63742.67 ± 3990.68 | 62942.00 ± 4080.98 |
| **51** | 2132.00 ± 281.13 ** | 73268.00 ± 4999.01 | 61368.33 ± 3827.01 * | 51393.67 ± 5605.39 ** | 38543.67 ± 7726.72 ** |
| **52** | 2132.00 ± 281.13 ** | 73268.00 ± 4999.01 | 65899.00 ± 2711.02 * | 62089.00 ± 5475.50 ** | 53650.00 ± 3463.12 ** |
| **Ibuprofen** | 2132.00 ± 281.13 ** | 73268.00 ± 4999.01 | 63530.33 ± 4994.17 | 60789.67 ± 4489.31 * | 36894.67 ± 6013.18 ** |

** $p < 0.01$, * $p < 0.05$ *versus* LPS-treated group. Data presented is the mean ± S.D. of samples run in triplicate.
